# Supplementary material for: Pharmacokinetic and pharmacodynamic characterization of CD8-targeted lentiviral vector for in vivo CD19-directed CAR-T therapy
Source: Mol Ther Adv. 2026 Jul 3;34(3):201803. doi: 10.1016/j.omta.2026.201803 (PMC13400396; doi:10.1016/j.omta.2026.201803)
Supplement: Document S1. Figures S1–S4 and Tables S1 and S2 [file mmc1.pdf]

OMTA, Volume *34*

Supplemental information

Pharmacokinetic and pharmacodynamic characterization of CD8-targeted lentiviral vector for *in vivo* CD19-directed CAR-T therapy

Victoria Duback, Shalu S. Kharkwal, Vasily Vagin, Kerrie Paterson, Alicia Sabbio, Joohwan Kim, Amey Gaikwad, Jesse Green, Brian Dolinski, Kelan Hlavaty, Garrett Zipp, Shannon Joyce, Abigail Koppes, Bindu Varghese, Kyle Trudeau, Jagesh Shah, Terry Fry, and Kutlu G. Elpek

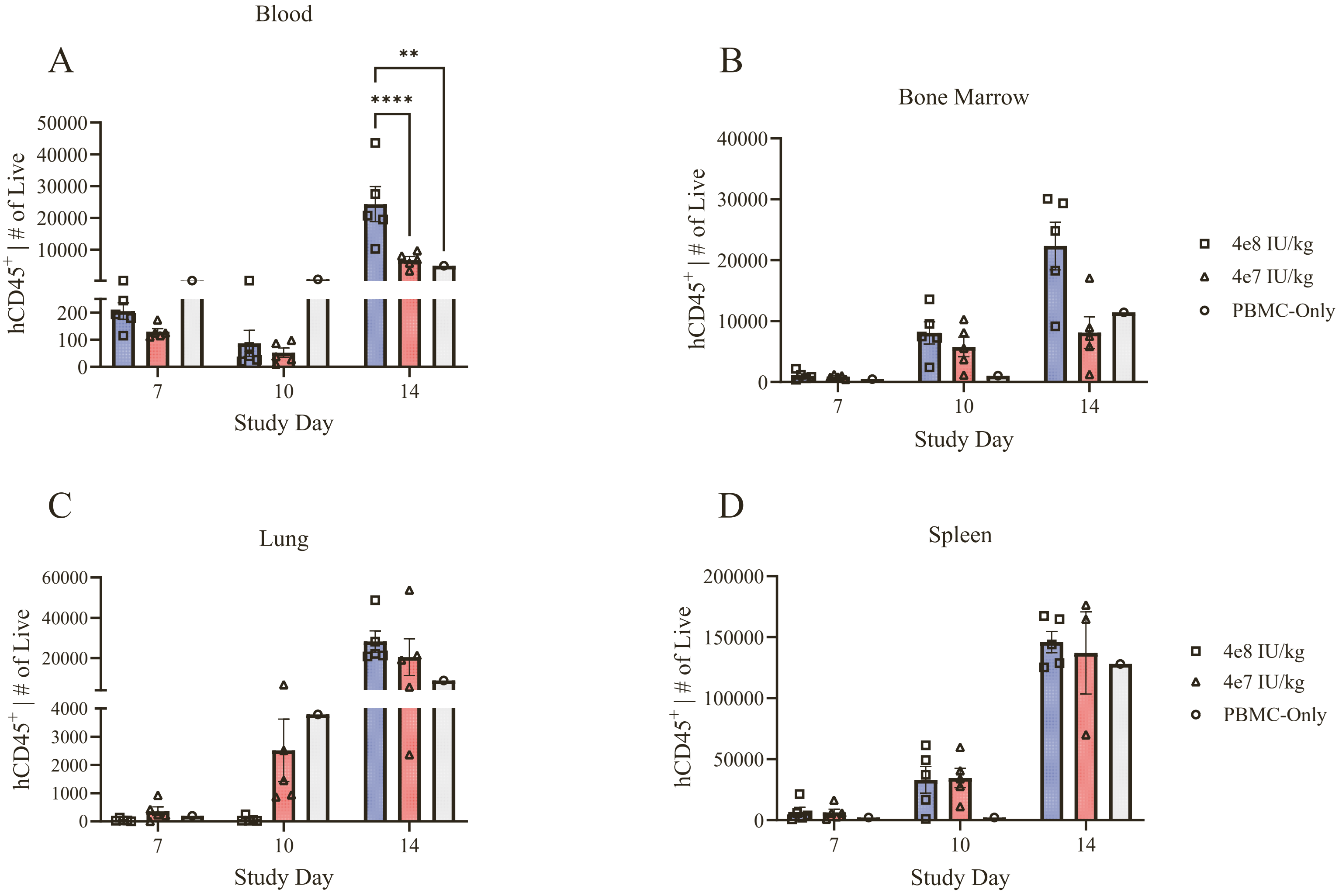
Blood
A
**
****
hCD45+ | # of Live
Study Day
B
Bone Marrow
hCD45+ | # of Live
Study Day
4e8 IU/kg
4e7 IU/kg
PBMC-Only
C
Lung
hCD45+ | # of Live
Study Day
D
Spleen
hCD45+ | # of Live
Study Day
4e8 IU/kg
4e7 IU/kg
PBMC-Only

Figure S1: Human CD45 Cell Detection in PBMC-engrafted Animals

Over Time

A) Number of $CD45^+$ cells per 100 µl of blood over time in PBMC-alone (black), 4e8 IU/kg (blue), and 4e7 IU/kg (red) of LVV. **B)** Number of $CD45^+$ cells per 100 µl of bone marrow over time in PBMC-alone (black), 4e8 IU/kg (blue), and 4e7 IU/kg (red) of LVV. **C)** Number of $CD45^+$ cells per 100 µl of lung over time in PBMC-alone (black), 4e8 IU/kg (blue), and 4e7 IU/kg (red) of LVV. **D)** Number of $CD45^+$ cells per 100 µl of spleen over time in PBMC-alone (black), 4e8 IU/kg (blue), and 4e7 IU/kg (red) of LVV. Female NSG mice (n=5/sex/group) engrafted with human PBMCs on day -1, and LVV on day 0. The LOD of the assay is > 20 events. Statistical significance was measured by calculating a two-way ANOVA with a Bonferroni multiple comparison (*p<0.05, **p<0.01, ***p<0.005, ****p<0.001).

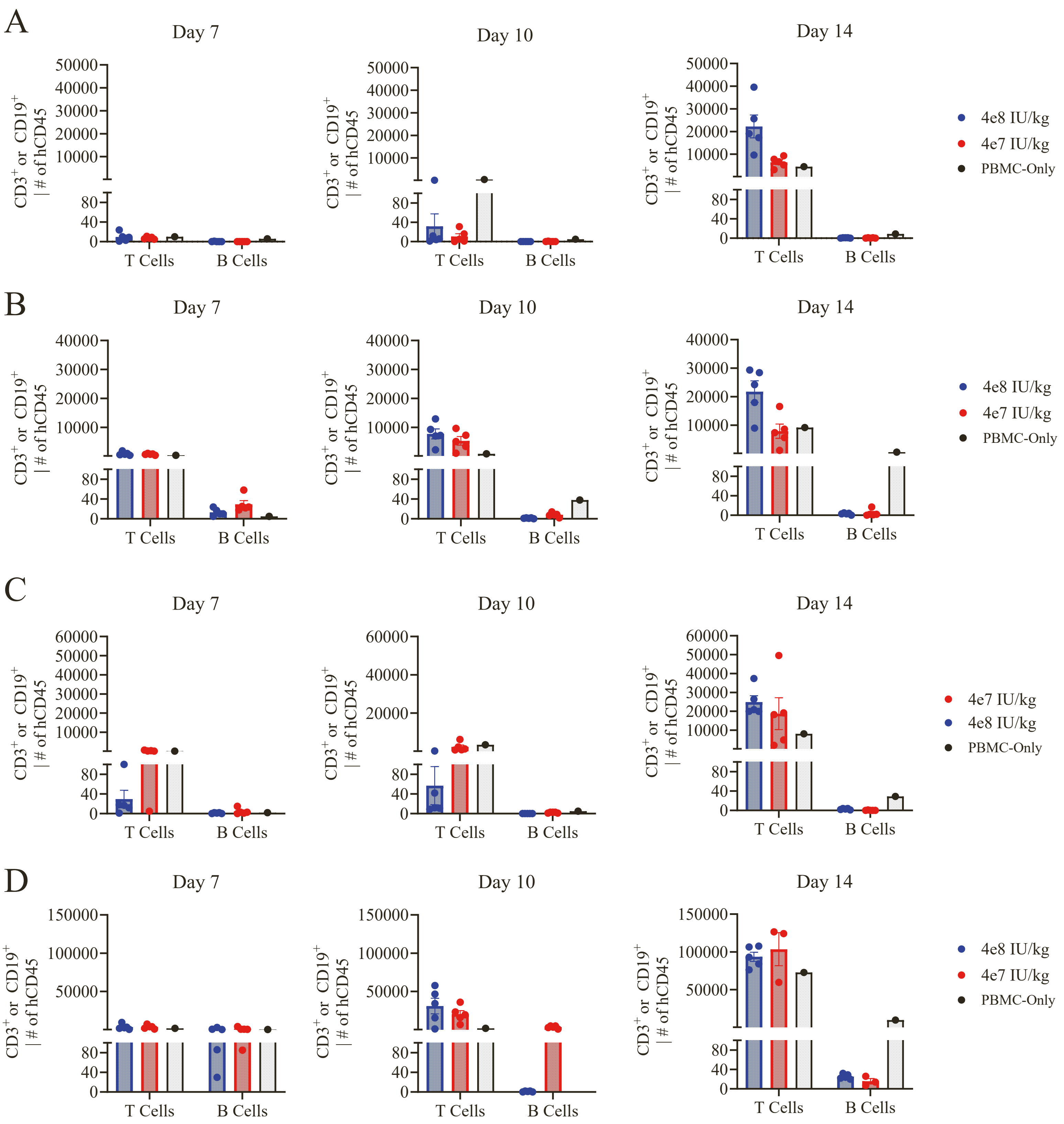
A
Day 7
Day 10
Day 14
CD3+ or CD19+ | # of hCD45
T Cells
B Cells
4e8 IU/kg
4e7 IU/kg
PBMC-Only
B
Day 7
Day 10
Day 14
CD3+ or CD19+ | # of hCD45
T Cells
B Cells
4e8 IU/kg
4e7 IU/kg
PBMC-Only
C
Day 7
Day 10
Day 14
CD3+ or CD19+ | # of hCD45
T Cells
B Cells
4e7 IU/kg
4e8 IU/kg
PBMC-Only
D
Day 7
Day 10
Day 14
CD3+ or CD19+ | # of hCD45
T Cells
B Cells
4e8 IU/kg
4e7 IU/kg
PBMC-Only

Figure S2: Human T Cell and B Cell Detection in PBMC-engrafted Animals Over Time

A) Number of $CD45^+$ $CD3^+$ and $CD19^+$ cells per 100 µl of blood on day 7, 10, and 14 in PBMC alone (black), 4e8 IU/kg (blue), and 4e7 IU/kg (red) of LVV. **B)** Number of $CD45^+$ $CD3^+$ and $CD19^+$ cells per 100 µl of bone marrow on day 7, 10, and 14 in PBMC-alone (black), 4e8 IU/kg (blue), and 4e7 IU/kg (red) of LVV. **C)** Number of $CD45^+$ $CD3^+$ and $CD19^+$ cells per 100 µl of lung on day 7, 10, and 14 in PBMC-alone (black), 4e8 IU/kg (blue), and 4e7 IU/kg (red) of LVV. **D)** Number of $CD45^+$ $CD3^+$ and $CD19^+$ cells per 100 µl of spleen on day 7, 10, and 14 in PBMC alone (black), 4e8 IU/kg (blue), and 4e7 IU/kg (red) of LVV. Female NSG mice (n=5/sex/group) engrafted with human PBMCs on day -1, and LVV on day 0. The LOD of the assay is > 20 events.

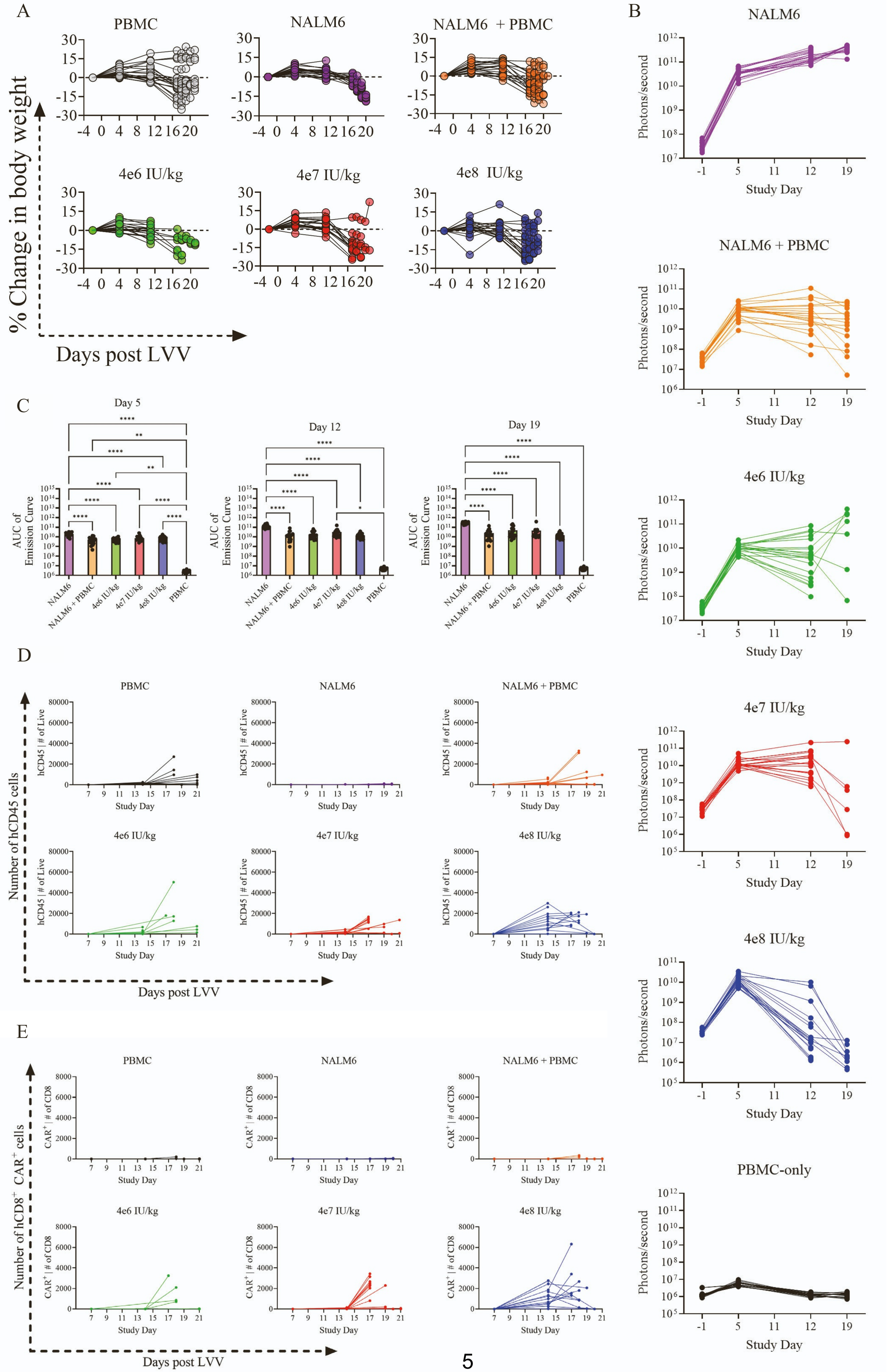
A
PBMC
NALM6
NALM6 + PBMC
4e6 IU/kg
4e7 IU/kg
4e8 IU/kg
% Change in body weight
Days post LVV
B
NALM6
NALM6 + PBMC
4e6 IU/kg
4e7 IU/kg
4e8 IU/kg
PBMC-only
Photons/second
Study Day
C
Day 5
Day 12
Day 19
AUC of Emission Curve
NALM6
NALM6 + PBMC
4e6 IU/kg
4e7 IU/kg
4e8 IU/kg
PBMC
D
PBMC
NALM6
NALM6 + PBMC
4e6 IU/kg
4e7 IU/kg
4e8 IU/kg
hCD45 | # of Live
Study Day
Number of hCD45 cells
Days post LVV
E
PBMC
NALM6
NALM6 + PBMC
4e6 IU/kg
4e7 IU/kg
4e8 IU/kg
CAR+ | # of CD8
Study Day
Number of hCD8+ CAR+ cells
Days post LVV

Figure S3: Pharmacodynamic Data from Donor 1

A) Percent change of body weight (from baseline measurement) over time in PBMC (black), NALM6 (purple), PBMC and NALM6 (orange), 4e6 IU/kg (green), 4e7 IU/kg (red), and 4e8 IU/kg (blue) of LVV. **B)** Photon per second values (tumor burden) graphed over time of individual animals that received either NALM6 (purple), PBMC and NALM6 (orange), 4e6 IU/kg (green), 4e7 IU/kg (red), 4e8 IU/kg (blue) of LVV or PBMC (black). **C)** Area under the curve of average photons per second (±SEM) values (tumor burden) graphed over time in PBMC (black), NALM6 (purple), PBMC and NALM6 (orange), 4e6 IU/kg (green), 4e7 IU/kg (red), and 4e8 IU/kg (blue) of LVV through day 5, 12, and 19. It is important to note that most animals were euthanized before the day 19 timepoint, so the data should be interpreted with caution. **D)** Number of $CD45^+$ cells per 100 µl of blood over time in PBMC (black), NALM6 (purple), PBMC and NALM6 (orange), 4e6 IU/kg (green), 4e7 IU/kg (red), and 4e8 IU/kg (blue) of LVV. The LOD of the assay is > 20 events. **E)** Number of $CD8^+CAR^+$ cells per 100 µl of blood over time in PBMC (black), NALM6 (purple), PBMC and NALM6 (orange), 4e6 IU/kg (green), 4e7 IU/kg (red), and 4e8 IU/kg (blue) of LVV. The LOD of the assay is > 20 events. Statistical significance was measured by calculating the area under the curve and measuring significance with a one-way ANOVA with a Bonferroni multiple comparison (*p<0.05, **p<0.01, ***p<0.005, ****p<0.001). Male and female NSG mice (n=5/sex/group) engrafted with $CD19^+$ NALM6 tumor cells on day -3, human PBMCs on day -1 from donor 1, and LVV on day 0.

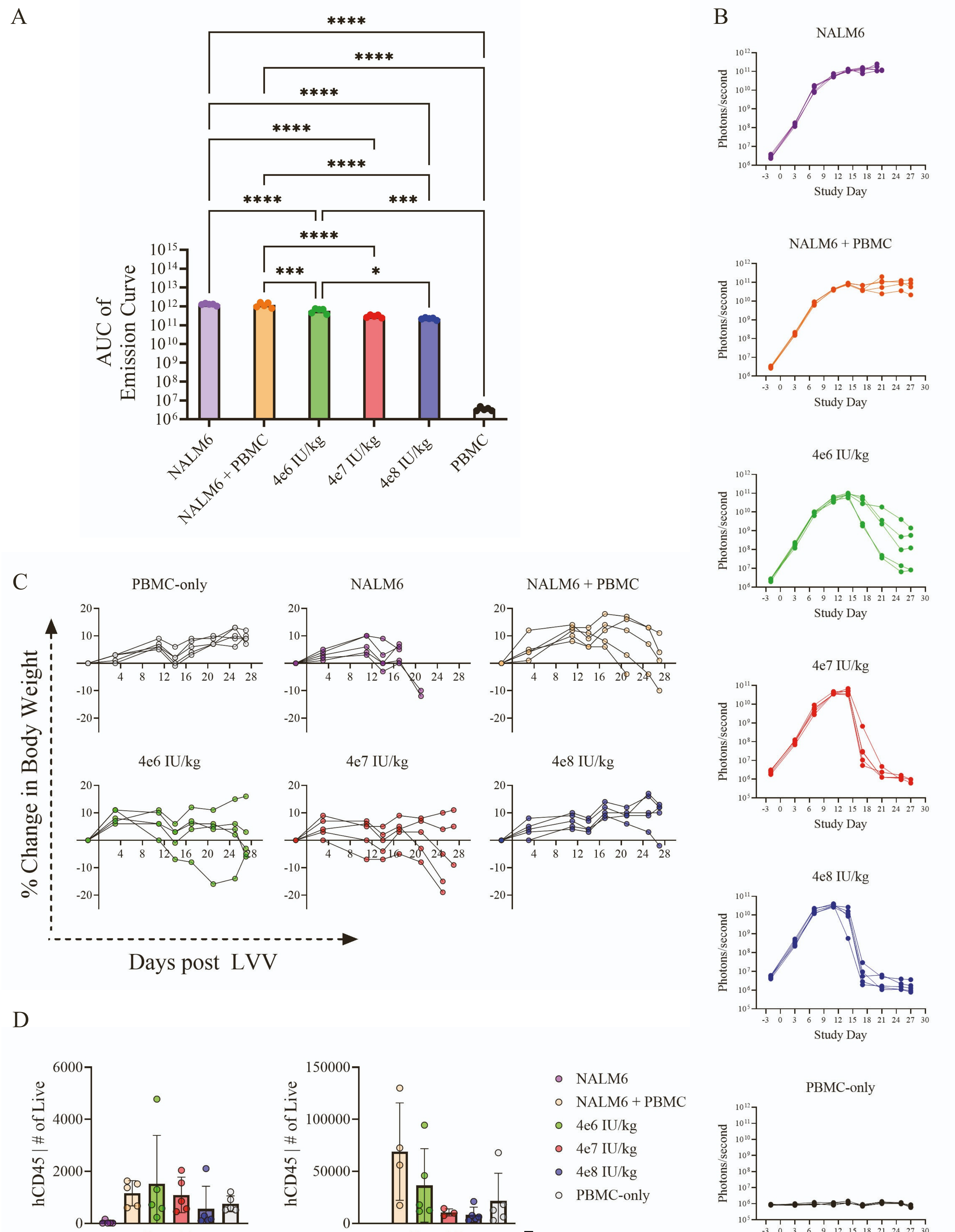
A
****
****
****
****
****
****
***
****
***
*
AUC of Emission Curve
NALM6
NALM6 + PBMC
4e6 IU/kg
4e7 IU/kg
4e8 IU/kg
PBMC
B
NALM6
NALM6 + PBMC
4e6 IU/kg
4e7 IU/kg
4e8 IU/kg
PBMC-only
Photons/second
Study Day
C
PBMC-only
NALM6
NALM6 + PBMC
4e6 IU/kg
4e7 IU/kg
4e8 IU/kg
% Change in Body Weight
Days post LVV
D
hCD45 | # of Live
Day 13
Day 27
NALM6
NALM6 + PBMC
4e6 IU/kg
4e7 IU/kg
4e8 IU/kg
PBMC-only

Figure S4: Pharmacodynamic Data from Donor 2

A) Area under the curve of average photons per second (±SEM) values (tumor burden) graphed over time in PBMC (black), NALM6 (purple), PBMC and NALM6 (orange), 4e6 IU/kg (green), 4e7 IU/kg (red), and 4e8 IU/kg (blue) of LVV over time. **B)** Photon per second values (tumor burden) graphed over time of individual animals that received either NALM6 (purple), PBMC and NALM6 (orange), 4e6 IU/kg (green), 4e7 IU/kg (red), 4e8 IU/kg (blue) of LVV or PBMC (black). **C)** Percent change of body weight (from baseline measurement) over time in PBMC (black), NALM6 (purple), PBMC and NALM6 (orange), 4e6 IU/kg (green), 4e7 IU/kg (red), and 4e8 IU/kg (blue) of LVV. **D)** Number of $CD45^+$ cells per 100 µl of blood over time in PBMC (black), NALM6 (purple), PBMC and NALM6 (orange), 4e6 IU/kg (green), 4e7 IU/kg (red), and 4e8 IU/kg (blue) of LVV. The LOD of the assay is > 20 events. Male and female NSG mice (n=5/sex/group) engrafted with $CD19^+$ NALM6 tumor cells on day -3, human PBMCs on day -1 from donor 2, and LVV on day 0. Statistical significance was measured by calculating the area under the curve and measuring significance with a one-way ANOVA with a Bonferroni multiple comparison (*p<0.05, **p<0.01, ***p<0.005, ****p<0.001).

Table S1. GQA Primer Sequences

Oligo Name	Primer and Probe Sequences
Viral Primer_Fwd	5'-GAGGAGGAGATATGAGGGACAA-3')
Viral Primer_Rev	5'-CACTCTTCTCTTTGCCTTGGT-3'
Viral Detection Probe	Fluorophore PrimeTime® 5`6-carboxyfluorescein (FAM™)<br>Non-fluorescent /ZENTM/and 3` Iowa Black® FQ(3`IABkFQ) double quencher:<br>5'- /5HEX/ATTGAACCA/ZEN/TTAGGAGTAGCACCC/3IABkFQ/-3'
mKate2_Fwd	5'-GTGGACAGAAGACTGGAAAGAA-3'
mKate2_Rev	5'-CTAGGGAGGTCGCAGTATCT-3'
mKate2 Detection Probe	Fluorophore PrimeTime ® 5`hexochlorofluorecein (HEX™)<br>Non-fluorescent /ZENTM/and 3` Iowa Black® FQ (3`IABkFQ) double quencher<br>5'-/5HEX/TCAAGGAGG/ZEN/CCGACAAAGAGACCTA/3IABkFQ/-3'

Table S2. VCN Primer Sequences

Oligo Name	Primer and Probe Sequence
CAR_Fwd	5'- CTCAAGAGGAAGATGGCTGTAG -3'
CAR_Rev	5'- CTGCTGAACTTCACTCTCAGT -3'
CAR_probe	5'- /56-FAM/CCTCCTTCT/ZEN/TCTTCTTCTGGAAATCGGC/3IABkFQ/- 3'
ARX_UCNE_Fwd	5'-TATGTTCAGATGCCCATTAGGG-3'
ARX_UCNE_Rev	5'-CTTGCTCAAAGGACTGTGATTTC-3'
ARX_UCNE_probe	5'-/5HEX/AGTGCCTTT/ZEN/CAGATGGAAACGGGT/3IABkFQ/-3'
