# Supplementary material for: Pharmacokinetic and pharmacodynamic characterization of CD8-targeted lentiviral vector for in vivo CD19-directed CAR-T therapy
Source: Mol Ther Adv. 2026 Jul 3;34(3):201803. doi: 10.1016/j.omta.2026.201803 (PMC13400396; doi:10.1016/j.omta.2026.201803)
Supplement: Document S2. Article plus supplemental information [file mmc2.pdf]

Molecular Therapy
Advances
Original Article

Pharmacokinetic and pharmacodynamic characterization of CD8-targeted lentiviral vector for *in vivo* CD19-directed CAR-T therapy

Victoria Duback,[1,2] Shalu S. Kharkwal,[1] Vasily Vagin,[1] Kerrie Paterson,[1] Alicia Sabbio,[1] Joohwan Kim,[1] Amey Gaikwad,[1] Jesse Green,[1] Brian Dolinski,[1] Kelan Hlavaty,[1] Garrett Zipp,[1] Shannon Joyce,[1] Abigail Koppes,[2] Bindu Varghese,[1] Kyle Trudeau,[1] Jagesh Shah,[1] Terry Fry,[1] and Kutlu G. Elpek[1]

[1]Sana Biotechnology, Inc., Cambridge, MA 02139, USA; [2]Northeastern University, Department of Chemical Engineering, Boston, MA 02115, USA

Given challenges with access, manufacturability, and the requirement for lymphodepletion with autologous chimeric antigen receptor (CAR)-T cell therapy, a promising alternative is *in vivo*-mediated gene delivery using redirected viral vectors to generate tumor-antigen-specific CAR-T cells. While data supporting *in vivo* CAR-T cells in patients are emerging, limited studies have described the pharmacokinetics (PK) and pharmacodynamics (PD) of this approach. Here, we evaluated the PK/PD of a novel CD8-targeted lentiviral vector called "fusosome," delivering a CD19-directed CAR transgene in xenograft mouse models. In NSG mice without target cells, the fusosome vector genomes cleared rapidly from plasma (90 minutes to 2 hours) and were detectable in tested tissues for up to 1 week. We observed prolonged persistence of viral particles in peripheral blood mononuclear cell (PBMC)-engrafted mice, with vector detection peaking within 10–40 min post-administration, depending on the tissue type. CAR transgene was detectable in various lymphoid organs after 7 days post-vector dosing. Dose-dependent tumor control and specific CAR-T cell generation were observed in tumor-bearing PBMC-engrafted mice. Overall, the PK/PD of fusosome and efficacy in the tumor model supports its potential as an *in vivo* gene delivery approach to treat patients with B cell lymphomas.

INTRODUCTION

Chimeric antigen receptor (CAR) T cell therapy has transformed the landscape of cancer treatment by enabling the precise targeting and elimination of malignant cells.[1–4] Despite its clinical success, conventional CAR-T therapy faces significant limitations, including the requirement for lymphodepleting chemotherapy, complex manufacturing logistics, and limited accessibility for many patients.[5–7] In response, alternative approaches are being developed to improve the scalability, efficacy, and safety of CAR-T cell therapy.[1,7,8]

Two such alternatives are allogeneic CAR-T cell therapy, which uses healthy-donor-derived T cells for off-the-shelf availability, and *in vivo*-generated CAR-T cell therapy, wherein T cells are engineered directly within the patient's body.[1,9–14] The latter approach holds promise for overcoming the logistical and manufacturing challenges of *ex vivo* CAR-T cells.[1,5,13] Importantly, *in vivo* CAR-T cell therapy simplifies manufacturing and eliminates the need for potentially harmful lymphodepleting chemotherapy.[11,13–16] *In vivo* CAR-T generation typically relies on viral or non-viral vectors to deliver the CAR transgene to T cells.[13,15–17] While non-viral methods offer advantages, such as simplicity of manufacturing, they suffer from off-target delivery, may require repeat dosing, and have transient gene expression, resulting in inconsistent therapeutic outcomes.[11,17,18] However, a recent study showed that mRNA/LNP approach could generate greater than 50% CAR$^+$ cells after each treatment cycle in lupus nephritis patients, resulting in B cell depletion and increasing confidence in non-viral approaches.[19]

Viral vectors, by contrast, enable sustained gene expression even in rapidly dividing cells via integration of the payloads (i.e., transgenes) into the genome. Recent developments in viral delivery platforms have leveraged fusogen proteins from enveloped viruses, which can be redirected to target specific cell types by ablating binding to their cognate receptors via specific mutations and then adding a binding moiety specific for the target cell type.[20,21] In particular, paramyxovirus fusogens fuse to cells in a pH-independent manner and have two envelope proteins that drive function: the fusion protein (F) and the receptor attachment protein (G).[20–22] Cell-specific targeting is achieved due to the separation of function between the fusion and receptor attachment proteins.[19] Such engineered fusogens have been used in pseudotyped lentiviral vectors to successfully and specifically transduce CD3$^+$, CD4$^+$, and CD8$^+$ T cells *in vivo*, demonstrating functional CAR expression and tumor-killing capacity in preclinical models.[23–27] However, pre-clinical and clinical data on vector

Received 17 November 2025; accepted 2 July 2026;
https://doi.org/10.1016/j.omta.2026.201803.

Correspondence: Kutlu G. Elpek, Sana Biotechnology, Inc., Cambridge, MA 02139, USA.
E-mail: kutlu.elpek@sana.com

Published by Elsevier Inc. on behalf of The American Society of Gene and Cell Therapy.
This is an open access article under the CC BY license (http://creativecommons.org/licenses/by/4.0/).

pharmacokinetics (PK), dose-dependent CAR-T cellular kinetics, biodistribution, and off-target effects remain limited and are essential to developing the field further, as these therapies are currently in first-in-human testing in cancer patients.[14,16,19,28–30] Early-phase clinical studies have demonstrated the feasibility of generating CAR-T cells *in vivo* in humans, with preliminary evidence of on-target B cell depletion and acceptable early safety profiles, while comprehensive efficacy and PK/PD data are still emerging.

The PK and biodistribution of viral vectors as drug products are expected to affect the magnitude of response and cellular kinetics of the *in vivo*-generated CAR-T cells. Therefore, it is critical to understand the relationship between the PK of the vector and its PD effects, including the cellular kinetics of the CAR-T cells. In this study, we evaluated the PK, biodistribution, specificity, and efficacy of a CD8-targeted vector (CD8/CD19CAR), called "fusosome," designed to generate $CD8^+$ CD19-directed CAR-T cells *in vivo* in xenograft mouse models. This fusosome system incorporates a self-inactivating (SIN) lentiviral vector scaffold with HIV packaging elements and a CD8-retargeted Nipah virus envelope modified to ablate its cognate receptor and selectively deliver a second-generation CD19-targeting CAR transgene to $CD8^+$ T cells. We evaluated the CD8/CD19CAR fusosome in naive and humanized NSG mouse models to understand the relationship between dose and PK in the presence or absence of the target cells and the pharmacodynamic effect in the presence of established tumors. These findings provide a critical foundation for continued development and clinical translation of fusosome-based CAR-T therapies and may inform future strategies targeting other T cell subsets.

RESULTS

Pharmacokinetics of a CD8-targeted fusosome in naive NSG mice

We first assessed CD8/CD19CAR fusosome clearance and biodistribution in the plasma of naive NSG mice in the absence of target cells (i.e., human $CD8^+$ T Cells). Animals were dosed intravenously (IV) with saline or fusosome at either 4e8 integrating units (IU)/kg (corresponding to ∼1e7 IU/25 g mouse; ∼6.08e10 vector genomes (vg)/25 g mouse) or 4e7 IU/kg (corresponding to ∼1e6 IU/25 g mouse; ∼3.04e9 vg/25 g mouse). Samples were collected in a time course (10–120 min) post-administration (Figure 1A), and vector genomes were measured via digital droplet polymerase chain reaction (ddPCR). The observed peak viral genome concentration (C_{max}) was observed in the plasma at 10 min, the first time point measured for both dose levels (Table 1; Figure 1B). Viral genomes were undetectable on average in the 4e8 IU/kg and 4e7 IU/kg dose groups by 120 min and 92 min (T_{last}), respectively, with a half-life ($T_{1/2}$) of 22.7 min for 4e8 IU/kg and 14.5 min for 4e7 IU/kg (Table 1). Area under the curve (AUC) analysis indicated that >90% of the vector was cleared after 30 min. In short, viral genome exposure in plasma, based on C_{max} and AUC_{0-t}, increased 25- and 35-fold, respectively, with a 10-fold increase in dose, suggesting a greater-than-dose-proportional increase in exposure with dose (Figure 1B).

Next, we ran a second cohort with only a 4e8 IU/kg (corresponding to ∼1e7 IU/25 g mouse; ∼3.51e10 vector genomes [vg]/25 g mouse) dose of fusosome to evaluate whether the vector was distributed to select tissues, including the liver, lung, and spleen (Figure 1C). The PK of fusosome in the plasma was overall similar, although the T_{last} was reduced to 90 min, indicating experiment-to-experiment variability in clearance between different lots of viruses (Figure 1D). Fusosome was detected in all tested tissues, and similar to plasma, C_{max} was at 10 min, the first time point measured, with extended exposure for up to 1-week post-administration (72 h in the liver, 1 week in the lung, and 72 h in the spleen; Figures 1E–1G). In both cohorts, the vector genomes in the plasma and tissues of control animals remained below the limit of detection (LOD).

Pharmacokinetics of CD8-targeted fusosome in PBMC-engrafted NSG mice

To understand the contribution of target-mediated clearance and biodistribution, we next used NSG mice that were pre-engrafted with PBMCs on study day 1, followed by the administration of either a 4e8 IU/kg (corresponding to ∼1e7 IU/25 g mouse; ∼6.08e10 vg/25 g mouse) or 4e7 IU/kg (corresponding to ∼1e6 IU/25 g mouse; ∼3.04e9 vg/25 g mouse) dose of fusosome on day 0. Vector genomes in the plasma and select tissues (spleen, lungs, and liver) were measured using ddPCR in a similar time course following fusosome administration (Figure 2A).

Peak viral genome concentration (C_{max}) in plasma for both dose levels was observed at the first post-dose time point (10 min) following administration of the fusosome (Figure 2B). Although the T_{last} in plasma was similar (90 min) to the cohort without target cells, the viral genome concentration (C_{last}) was 13-fold higher in 4e8 IU/kg-treated mice and 2.5-fold higher in 4e7 IU/kg-treated mice compared to the animals without target cells (Table 1; Table 2). AUC analysis indicated that >80% of the vector was cleared after 30 min for both dose levels. Contrary to mice without target cells, viral genome exposure in plasma in 4e8 IU/kg- and 4e7 IU/kg-treated mice, based on the C_{max} and AUC_{0-t}, increased 4.19- and 3.64-fold, respectively, implying a target-cell mediated increase in vector exposure in plasma and suggesting a less-than-dose-proportional increase in exposure with a 10-fold increase in the dose (Figure 2B). The elimination of viral particles appears to be multi-phasic in plasma, with an initial fast decline in viral concentration followed by a moderate decline from 30 to 90 min post-dose.

The C_{max} in the spleen, lung, and liver were also observed at the first post-dose time (10 min) in the 4e7 IU/kg group and ranged from 20 to 40 min post-dose in the 4e8 IU/kg group (Figures 2C and 2D; Table 2). The half-life of the vector extended to 3.75–12 h in 4e7 IU/kg-treated mice and 1.7–10.4 h in 4e8 IU/kg-treated mice (Table 2) in the presence of PBMCs. Furthermore, AUC analysis indicated that viral particle exposure of the first 90 min (AUC_{0-90}) was 53%–96% and 37%–88% of the total exposure measured in mice treated with 4e7 IU/kg or 4e8 IU/kg fusosome, respectively, suggesting that most of the viral exposure occurs in

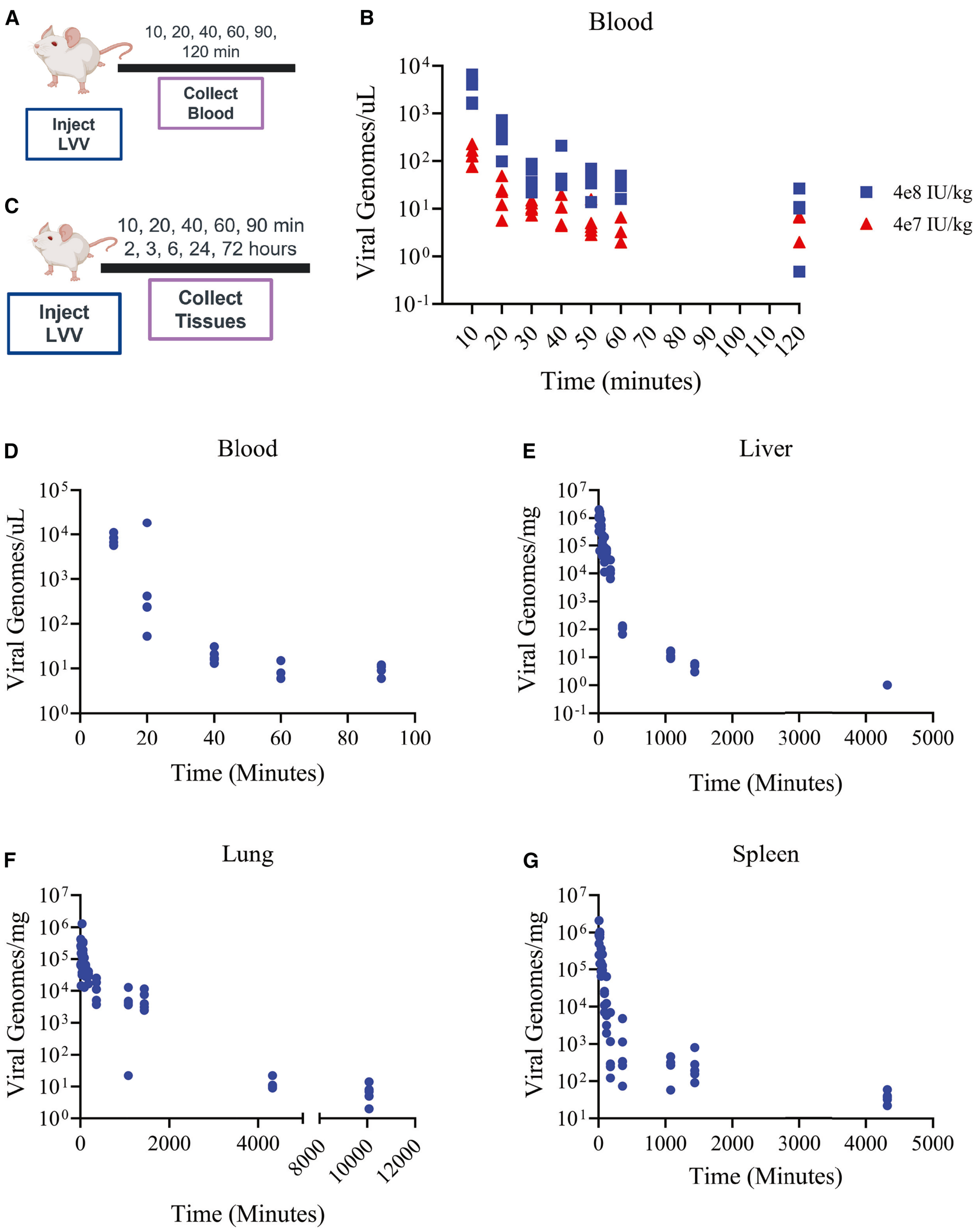

Figure 1. Dose-dependent clearance of fusosome in naive NSG mice

(A) Study design schematic of female NSG mice administered an LVV dose of 4e8 IU/kg or 4e7 IU/kg (n = 5/group) at time zero, and samples were collected from 10 to 120 min to measure viral genomes through a genome quantification assay (GQA). (B) Viral genomes per microliter of blood in mice dosed with a 4e8 IU/kg of LVV (blue squares) or 4e7 IU/kg (red triangles) over time (minutes). (C) Study design schematic of female NSG mice administered a 4e8 IU/kg of LVV or saline at time zero. Blood and tissues were collected from 10 min to 72 h to evaluate the clearance and distribution of viral particles over time. (D) Viral genomes per microliter of blood over time in naive NSG mice. (E) Viral genomes per microgram of liver tissue over time in naive NSG mice. (F) Viral genomes per microgram of lung tissue over time in naive NSG mice. (G) Viral genomes per microgram of spleen tissue over time in naive NSG mice. Only quantifiable values are reported in the graphs. Limit of detection (LOD) for this assay was $\geq$15 vector genomes per reaction. Lower limit of quantitation (LLOQ) for the assay was $\geq$60 vector genomes per reaction.

Table 1. Summary of PK parameters for naive NSG mice

Treatment	4e7 IU/kg IU/kg group	4e8 IU/kg IU/kg group
Analyte	viral particles	viral particles
$T_{½}$ (min)	14.5	22.7
C_{max} (viral genomes/μL)	143	3,660
T_{max} (min)	10.0	10.0
T_{last} (min)	92.0	120
C_{last} (viral genomes/μL)	4.75	11.6
AUC_{0-t} (min*viral genomes/μL)	5,630	202,000
AUC_{0-30} (min*viral genomes/μL)	5,270	199,000
AUC_{30-120} (min*viral genomes/μL)	391	2,660

Notes: all values were calculated using Phoenix WinNonlin v.8.3 (Certara USA, Inc., Princeton, New Jersey). Rsq values < 0.80 for T½ analysis are not considered acceptable but have been reported for completeness. LOD for this assay was ≥15 vector genomes per reaction. LLOQ for the assay was ≥ 60 vector genomes per reaction.

the first 90 min after dosing; however, there was variability in exposure between the tissues (Figures 2C–2E; Table 2). Viral genome exposure increased more than dose-proportional in all tissues, with the most significant increase in the spleen (Figures 2C–2E; Table 2). An initial distribution phase was observed, particularly at the higher dose level, followed by a moderate decline in viral particles (Figures 2C–2E; Table 2). The vector genomes in the plasma and tissues of control animals remained below the LOD throughout the time course.

Generation of CD19-targeted CAR-T cells in PBMC-engrafted NSG mice

Next, we evaluated whether the PK of the fusosome at these dose levels was sufficient to generate CAR-T cells using a similar model (Figure 3A). First, we measured the genome integration and biodistribution of the CAR transgene in target cells using ddPCR. CAR vector copy numbers (VCNs) were calculated as integrations per diploid genome on days 7, 10, and 14 post-administration of the fusosome and were reported as undetectable (i.e., below the limit of quantitation) or with a quantifiable signal value (Table 3).

In the 4e8 IU/kg-treated animals, quantifiable CAR VCN signal started to appear on day 7 post-administration, with the highest levels in the spleen, followed by the bone marrow and lung (0.0014, 0.0008, and 0.0002, respectively; Table 3). All other tissues had undetectable CAR signals except for a few instances at this time point (Table 3). The VCN signal increased gradually over time in tissues, indicating expansion of CAR^+ cells. On day 10, the CAR signal was quantifiable in the majority of the tissues, with the highest levels in the whole blood, spleen, lungs, and liver (0.1205, 0.0729, 0.0211, and 0.0154, respectively; Table 3). Quantifiable CAR signal was higher on day 14 compared with day 10 and was detected across both lymphoid and non-lymphoid tissues. The highest levels were observed in the spleen, whole blood, and lung, with lower but detectable levels in the bone marrow, liver, gonads, brain, kidneys, and heart (Figures 3B; Table 3).

On the other hand, consistent with dose levels, the overall CAR VCN signal was lower at all time points in animals that received the 4e7 IU/kg dose of fusosome. On day 7, most tissues in 4e7 IU/kg-treated mice had undetectable CAR signals, except a few animals in the spleen, lung, or liver (Table 3). By day 10, the VCN signal was highest in the spleen, followed by the lungs, bone marrow, and liver (0.0065, 0.0022, 0.0016, and 0.0011, respectively; Table 3). Similar to the high-dose groups, quantifiable CAR signal was detected in all tested tissues on day 14, with the highest signal observed in the whole blood and spleen (0.1479 and 0.0711, respectively; Table 3). All PBS-treated control animals had CAR VCN signals below the limit of detection in all tested tissues. Overall, these data demonstrate that the CAR signal increases with time in a dose-dependent manner, and CAR-T cells are distributed to tissues. However, it is important to note that human cell expansion is observed in all tissues over time in this mouse model, regardless of treatment with fusosome, which is a limitation of the model and likely caused by xeno-reactive graft-versus-host disease (Figure S1). The majority of these human $CD45^+$ cells are T cells. B cells in the PBMCs may contribute to CAR-T cell expansion; however, this is likely an early response, as B cells do not engraft well in this model (Figure S2).

Next, we analyzed CAR^+ $CD3^+$ T cells in the blood, bone marrow, lung, and spleen of PBMC-engrafted mice treated with either the 4e8 IU/kg or 4e7 IU/kg dose of fusosome using flow cytometry to evaluate CAR protein expression. CAR^+ T cells in the blood of treated animals were below the limit of detection (LOD; ≥20 events) on days 7 and 10 and below the LOD in tissues on day 7. The number of CAR-T cells increased over time in the blood (Figure 3B) and tissues (Figures 3C–3E), consistent with the VCN data. As with the VCN measurements, there was dose dependency, with more CAR-T cells generated in all tissues in the 4e8 IU/kg-treated mice compared to 4e7 IU/kg-treated animals (Figures 3B–3E). Collectively, these results indicate a dose-dependent, tissue-wide distribution of CAR^+ cells over time, consistent with the CAR transgene integration data.

Functional efficacy of *in vivo*-generated CAR-T cells in the presence of tumor cells

A similar model was used to assess the dose-dependent efficacy of *in vivo*-generated CAR-T cells in mice bearing $CD19^+$ NALM6 tumor cells administered intravenously 3 days before PBMC engraftment (Figure 4A). Several control groups were included in the study, including NALM6-only (tumor kinetics), PBMCs-only (engraftment control), and NALM6 + PBMC without fusosome (to control for alloreactive T cell-mediated anti-tumor effects). Fusosome treatment of 4e8 IU/kg (∼1e7 IU/25 g mouse; ∼3.51e10 vg/25 g mouse), 4e7 IU/kg (∼1e6 IU/25 g mouse; ∼1.76e9 vg/25 g mouse), or 4e6 IU/kg (∼1e5 IU/25 g mouse; ∼4.39e8 vg/25 g mouse) was administered to mice with established tumors and was well tolerated at all dose levels post-infusion. There were no changes in

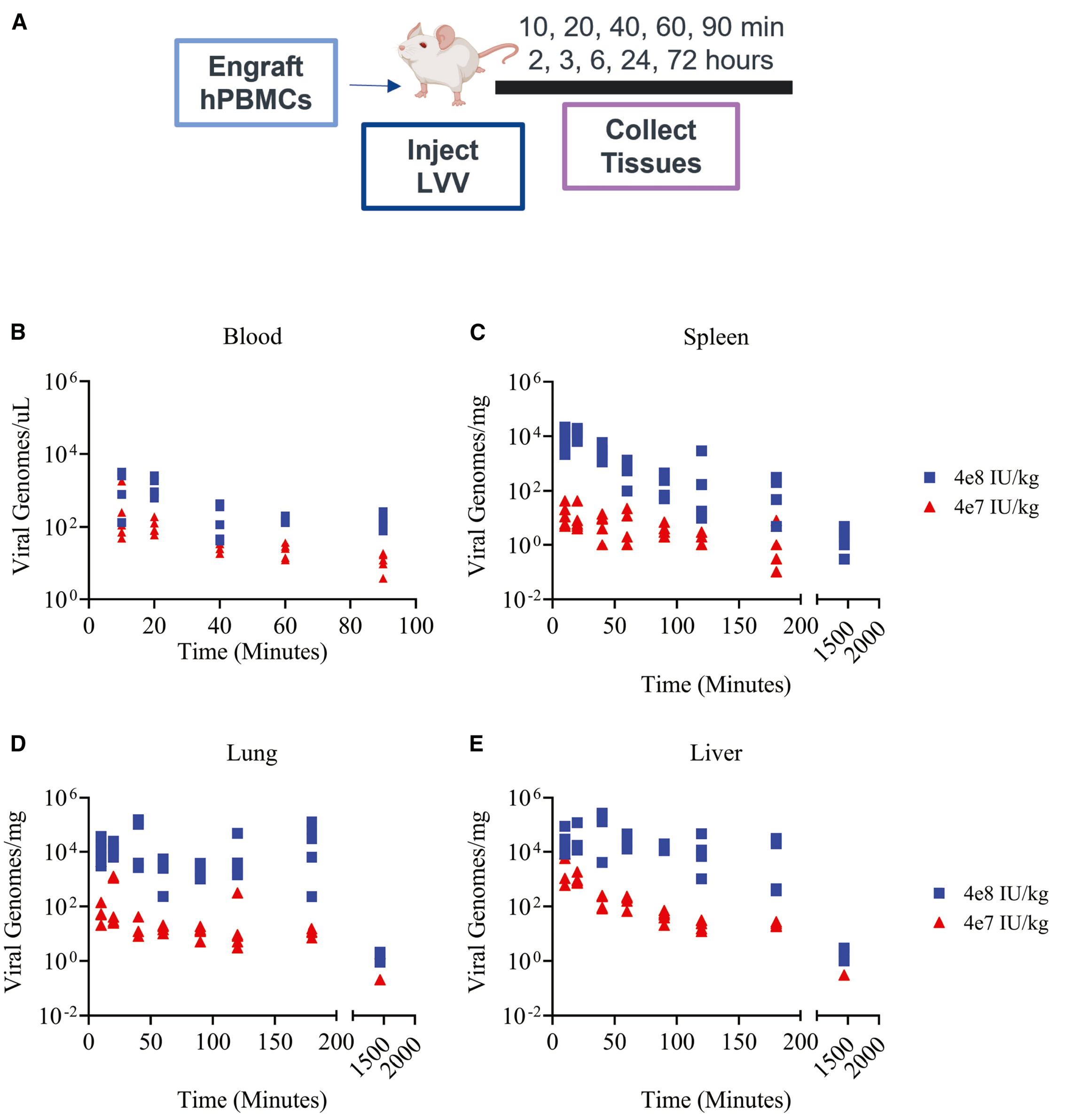

Figure 2. Clearance of CD8-targeted fusosome in PBMC-engrafted NSG mice

(A) Study design schematic of female NSG mice (n = 5/group) engrafted with human PBMCs (day 1) and LVV (day 0). Tissues and blood were collected 10, 20, 40, 60, and 90 min and 2, 3, 6, 24, and 72 h post-fusosome injection. Mice were dosed with 4e8 IU/kg of LVV (blue squares) or 4e7 IU/kg (red triangles). (B) Viral genomes per microliter of blood in mice over time (minutes). (C) Viral genomes per microgram of spleen tissue in mice over time. (D) Viral genomes per microgram of lung tissue in mice over time. (E) Viral genomes per microgram of liver tissue in mice over time. Only quantifiable values are reported in the graphs. LOD for this assay was ≥15 vector genomes per reaction. LLOQ for the assay was ≥60 vector genomes per reaction.

body weight related to fusosome treatment compared to control groups up to 12 days post-infusion (Figure S3A).

In this study, complete tumor control was observed in the 4e8 IU/kg and 4e7 IU/kg treatment groups relative to the NALM6 only group, as assessed by longitudinal AUC analysis of tumor burden and endpoint analysis using a linear mixed effects model at day 19 post-fusosome administration (Figure 4B; Figures S3B and S3C). AUC analysis revealed early dose-dependent differences in tumor suppression; however, by day 19, cumulative tumor burden did not differ between treatment groups (Figure S3C). In contrast, linear mixed effects modeling (LME) of tumor burden at day 19 demonstrated a significant reduction in tumor burden in the 4e8 IU/kg group compared to both the NALM6only and

NALM6 + PBMC control groups, as well as a significant difference between the 4e8 IU/kg and 4e6 IU/kg dose groups (Figure 4B). No statistically significant differences were observed between the 4e8 IU/kg and 4e7 IU/kg dose groups at this time point. Notably, the PBMC donor used in this study exhibited marked tumor control (NALM6+PBMC) relative to NALM6-only (AUC and LEM), consistent with a potential allogeneic antitumor response, which complicates interpretation of CART-mediated tumor control in this model (Figure 4B; Figures S3B and S3C).

Analysis of day 14 peripheral blood samples showed CAR^+ cell frequencies in a dose-dependent manner (16× higher in the 4e8 IU/kg vs. 4e7 IU/kg and 47× higher in 4e8 IU/kg vs. 4e6 IU/kg) in CD8 T cells (Figure 4C). CAR expression remained undetectable in off-target $CD8^-CD4^+$ T cells across all treated groups, supporting the specificity of the fusosome (Figure 4C). Human $CD45^+$ cell expansion in the first 14 days was more pronounced in fusosome-treated animals, particularly at the highest dose, and over time, $CD45^+$ cells (Figure S3D) and $CD8^+CAR^+$ cells (Figure S3E) continued to expand in all groups engrafted with PBMCs. Human cell expansion, decrease in body weight, and high morbidity observed in all PBMC-engrafted groups after day 12 are likely driven by xeno-reactive graft-versus-host disease (Figure S3).

Given the apparent influence of allo- and xeno-reactivity on tumor control at intermediate and low fusosome doses, dose-dependent efficacy was further evaluated using an additional PBMC donor. Consistent with the prior study, treated animals exhibited a significant reduction in tumor burden compared to the NALM6-only group across all dose levels (Figure 4D; Figure S4A). Longitudinal AUC analysis of tumor burden demonstrated a significant decrease in tumor burden in all treatment groups relative to NALM6-only controls (Figure S4A), with dose-dependent differences observed between the 4e8 IU/kg and 4e6 IU/kg groups. In contrast to the first PBMC donor, AUC analysis also revealed significantly lower tumor burden in all treated groups compared to the NALM6 + PBMC control group (Figure S4A). Endpoint analysis using a linear mixed effects model at day 27 similarly showed significant reductions in tumor burden for all treated groups relative to the NALM6 + PBMC control, independent of dose (Figure 4D; Figure S4B), suggesting reduced allo- and xeno-reactive contributions to tumor control with the second PBMC donor.

Similar to the previous donor, dose-dependent CAR expression was detected only in $CD8^+$ T cells on day 13 post-administration (Figure 4D). CAR expression was still specific to $CD8^+$ T cells after 27 days, but the on-target expression level was similar for all doses by this time point (Figure 4F). Similar to the study above, fusosome administration was well tolerated across all dose levels, with no treatment-related changes in body weight observed compared to control groups through 12 days post-infusion (Figure S4C). Human $CD45^+$ cell expansion was observed within the first 13 days in all animals receiving PBMCs and continued to increase through day 27 in all PBMC-engrafted groups, regardless of fusosome dose (Figure S4D). Overall, human $CD45^+$ cell levels at day 27 were higher with the second PBMC donor compared to the first donor; however, based on the absence of treatment-related body weight changes and the observed differences in tumor suppression patterns, the contribution of allo- and xeno-reactive responses to tumor control was likely reduced with this donor PBMC.

DISCUSSION

In vivo-generated CAR-T cell therapy represents a promising strategy to overcome the limitations of traditional *ex vivo* manufacturing. Importantly, clinical trials using viral vectors for *in vivo*-generated CAR-T cells have been initiated in the last year with promising early results.[14,28–30] However, there is limited data characterizing the PK and PD of these therapies. In this study, we characterized the PK, biodistribution, and PD of a CD8-targeted fusosome designed to generate CD19-directed CAR-T cells *in vivo*. Our data show rapid systemic clearance of the vector in naive NSG mice and prolonged persistence in PBMC-engrafted mice, with vector detection peaking within 10–40 min post-administration, depending on the tissue type. Despite rapid clearance, the fusosome effectively transduced $CD8^+$ T cells, resulting in dose-dependent CAR expression, expansion, and anti-tumor activity.

Peak concentrations in vector genomes in the blood and tissues occurred within minutes of administration, with a dose-dependent decline over 72 h. Clearance was slower in PBMC-engrafted mice, likely due to viral engagement with target $CD8^+$ T cells, which delays systemic elimination. A potential reason for this observation is that $CD8^+$-T-cell-bound viral particles persist longer in the circulation and tissues, which in turn temporarily prevents rapid clearance, leading to a longer detection in plasma. NSG mice, which lack key immune clearance mechanisms, may not fully model human fusosome PK. Further studies in immunocompetent mice or non-human primates (NHPs) are necessary to understand the role of complement, phagocytic cells, and other factors on fusosome clearance and distribution. However, challenges remain in performing such translational studies, such as species differences in vector receptor binding and transduction efficiency, which limit the utility of standard murine models and NHPs, necessitating surrogate constructs.[31–34]

Viral vectors used for systemic gene delivery exhibit distinct PK/PD paradigms that are inherently platform dependent. For AAV, pharmacokinetic interpretation is largely driven by tissue biodistribution and persistence of vector genomes and transgene expression, rather than classical clearance alone, reflecting long-term episomal maintenance following a single administration.[32,35,36] AAV clearance kinetics are highly serotype dependent. For instance, van Gestel et al. reported blood half-lives ranging from approximately 4.2 min to 11 h across different serotypes following systemic administration in rats.[37] In contrast, lentiviral vectors are integrating platforms in which intact vector exposure is transient, and durable pharmacodynamic effects arise from target cell engagement, genomic integration, and subsequent expansion and trafficking of transduced cells.[38,39]

Table 2. Summary of PK parameters for PBMC-engrafted NSG mice

Matrix	Plasma		Matrix	Liver		Lung		Spleen	
Treatment	4e7 IU/kg	4e8 IU/kg	treatment	4e7 IU/kg	4e8 IU/kg	4e7 IU/kg	4e8 IU/kg	4e7 IU/kg	4e8 IU/kg
$T_{½}$ (min)	16.3	20.5	$T_{½}$ (min)	225	100	683	276	726	625
C_{max} (viral genomes/μL)	465	1,950	C_{max} (viral genomes/mg)	4,770	164,000	916	78,600	16.7	14,100
T_{max} (min)	10.0	10.0	T_{max} (min)	10.0	40.0	10.0	40.0	10.0	20.0
T_{last} (min)	90.0	90.0	T_{last} (min)	1,440	1,440	4,320	4,320	4,320	4,320
C_{last} (viral genomes/μL)	12.0	153	C_{last} (viral genomes/mg)	0.345	1.65	0.113	0.409	0.0297	0.187
AUC_{0-t} (min*viral genomes/μL)	16,500	60,100	AUC_{0-t} (min*viral genomes/mg)	161,000	6,570,000	30,300	4,880,000	1,700	469,000
AUC_{0-30} (min*viral genomes/μL)	14,000	48,500	AUC_{0-90} (min*viral genomes/mg)	155,000	4,780,000	23,100	1,790,000	900	412,000
AUC_{0-360} (min*viral genomes/mg)	2,520	11,500	AUC_{0-360} (min*viral genomes/mg)	159,000	6,560,000	27,400	4,860,000	1,280	458,000

Notes: all values calculated using Phoenix WinNonlin v.8.3 (Certara USA, Inc., Princeton, New Jersey). Rsq values < 0.80 for T½ analysis are not considered acceptable but have been reported for completeness. LOD for this assay was ≥15 vector genomes per reaction. LLOQ for the assay was ≥60 vector genomes per reaction.

Consistent with prior intravenous LV studies with VSVg-pseudotyped vectors, which show rapid vector clearance with early enrichment in reticuloendothelial and hematopoietic compartments,[40,41] CD8-targeted fusosome vector genomes declined rapidly from plasma with only transient tissue detectability. In contrast, CAR VCN and CAR^+ $CD8^+$ Tcell expansion increased over subsequent days, aligning with published *in vivo* LV-mediated CART generation despite brief systemic vector exposure.[24,27,42,43] Two recent clinical studies reported PK for an *in vivo*-delivered lentiviral CAR therapy in humans.[29,30] Presence of vector in circulation was analyzed at limited time points post-infusion, showing peak around 6–12 h followed by clearance by 48 h in one study,[30] and detectable vector on day 2 followed by clearance by day 4 in second study[29] despite receiving the same dose (2e8 TU/person). The difference in PK profile for this vector in humans and our vector in mouse models underscore that vector PK and biodistribution may differ substantially across species and delivery platforms and highlight the need for further studies to better understand how vector design, target cell populations, and host-specific factors influence *in vivo* CART cell kinetics and exposure in humans.

It is important to note that our study focused primarily on pharmacokinetics and did not evaluate clearance mechanisms (e.g., hepatic) or viral shedding in excreta. Viral shedding is an informative measurement to understand the possibility of transmission to other individuals.[32] As most viral vector *in vivo* therapies use replication-incompetent vectors, the safety risk of viral shedding is low.[32] While AAV clearance has been assessed in multiple body fluids (e.g., saliva, feces, and urine), blood-based measurements have generally been the most informative, reflecting higher and more robustly quantifiable vector genome levels.[44] Additionally, replication incompetent lentiviral vectors exhibit rapid clearance of intact vector from circulation with only minimal and transient detection in excreta, supporting plasma based PK as the primary and sufficient measure of vector clearance for lentiviral platforms.[43,45] Additionally, recent clinical data have shown no detection of viral particles in excreta over time in patients who received a lentiviral vector for *in vivo* CAR-T therapy.[29,30] However, additional experiments measuring viral shedding from urine and/or feces in an immunocompetent animal model could be informative as a mechanism to further characterize this fusosome.

Despite rapid clearance of vector genomes from circulation and tissues, we observed efficient CAR transgene integration into $CD8^+$ T cells within days of administration. CAR VCN increased in a dose-dependent manner between days 7 and 14, consistent with efficient vector integration and CAR T cell expansion. As noted before, the model shows the differences in CAR-T cell generation in a dose-dependent manner; however, CAR-T cell expansion is potentially driven by xeno-reactive response over expansion driven by low levels of B cells engrafted in the model. Correspondingly, CAR protein expression in circulation, lymphoid tissues, and bone marrow rose over the same period, demonstrating successful transcription and translation of the transgene. These data support the notion that brief vector exposure is sufficient for effective gene delivery, provided target cells are available for engagement. It is important to note that CAR levels were assessed in non-perfused tissues, which may increase the contribution of circulating blood to measured signal, particularly in non-lymphoid tissues at day 14. The observed increase in CAR VCN signal across all evaluated tissues by day 14, irrespective of lymphoid classification, is consistent with CAR-T cell expansion detected in peripheral blood and select tissues by flow cytometry in this model. In xenograft systems, human T cells are known to undergo broad expansion and trafficking into murine tissue over time as a result of xeno-reactive processes, independent of treatment modality.[46,47]

Functional CAR-T cell generation was observed across multiple PBMC donors, with robust, dose-dependent expansion of $CD8^+CAR^+$ T cells by day 14 post-administration and eradication of $CD19^+$ cells. CAR expression remained specific to $CD8^+$ T cells, with no off-target transduction in $CD4^+$ populations, confirming the targeting fidelity of the fusosome. CAR-T cell activity resulted

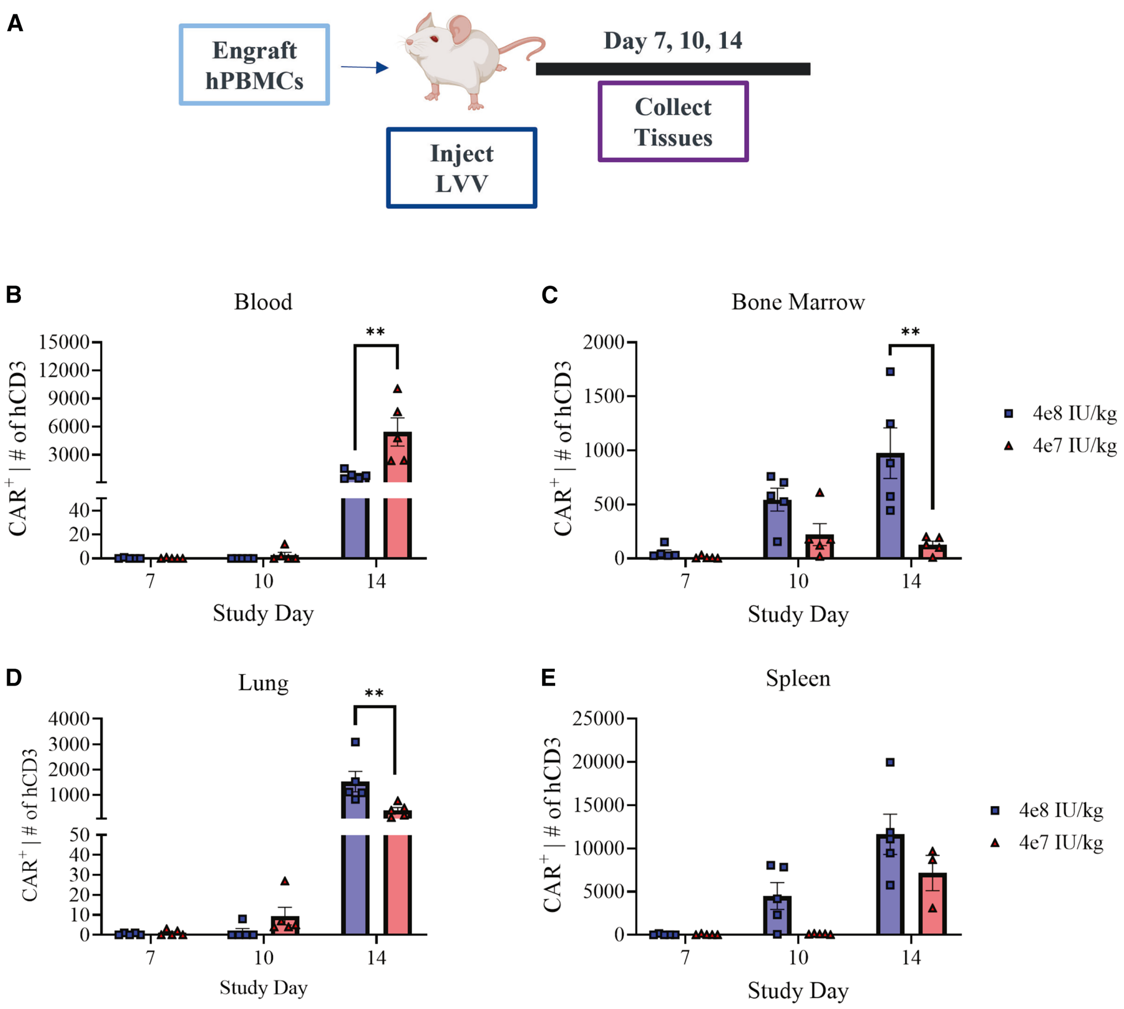

Figure 3. Biodistribution and genome integration of CAR by flow cytometry

(A) Study design schematic of female NSG mice (n = 5/group) engrafted with human PBMCs (day 1) and LVV on day 0. Tissues and blood were collected 7, 10, or 14 days post-fusosome injection. Mice were dosed with 4e8 IU/kg of LVV or 4e7 IU/kg. (B) Number of CAR^+ $CD3^+$ cells in the blood over time. (C) Number of CAR^+ $CD3^+$ cells in the bone marrow over time. (D) Number of CAR^+ $CD3^+$ cells in the lungs over time. (E) Number of CAR^+ $CD3^+$ cells in the spleen over time. Animals treated with a 4e8 IU/kg of LVV (blue squares) or 4e7 IU/kg (red triangles). The LOD of the assay was >20 events. Statistical significance was measured by calculating a two-way ANOVA with a Bonferroni multiple comparison (*$p < 0.05$, **$p < 0.01$, ***$p < 0.005$, ****$p < 0.001$). Error bars represent standard error of the mean (SEM).

in tumor suppression, even in the presence of donor-to-donor variability in CAR expression and alloreactivity. Donor-to-donor variability is a known feature of *ex vivo* CAR T cells and will be important to understand in the context of *in vivo* delivery. Differences in tumor control observed between PBMC donors at intermediate dose levels are likely influenced by donor-dependent alloreactivity, engraftment efficiency, and intrinsic T cell fitness rather than fusosome functionality. Similar donor-to-donor variability has been widely reported for *ex vivo* CAR-T products and represents a known limitation of xenograft models.[25,26,48] A limitation of the NSG xenograft model is the presence of allogeneic and xenogeneic immune responses, which confound the interpretation of CAR-T cell biodistribution and CAR-specific efficacy.[26,49–51] For example, the increase in VCN signal and CAR-T cells in mice without tumors may be due to both the trafficking of CAR-T cells into tissues and the overall expansion of T cells due to the xenoreactivity of human PBMCs in this mouse model. Similar patterns have been seen by others, where this artificial environment leads to the expansion and trafficking of CAR-T cells to off-target tissues over time.[52,53] However, we and others have demonstrated more restricted cell trafficking and limited biodistribution in autologous models, such as non-human primates (NHPs).[23,54,55] The selection of PBMC donors and the use of MHC class I/II knockout models could mitigate these issues and demonstrate that CAR-mediated tumor suppression persists even with reduced alloreactivity and xenoreactivity.[56,57]

Table 3. Biodistribution and genome integration of CAR by VCN

Day	Dose	Animal ID	SPLN	BM	LNG	WB	Liv	GON	KID	BR	HT
Day 7	PBS	GrpA-2	undetectable	undetectable	undetectable	undetectable	undetectable	undetectable	undetectable	undetectable	undetectable
	4e7 IU/kg	GrpB-51	detectable	detectable	0.0019	detectable	detectable	detectable	undetectable	undetectable	undetectable
		GrpB-52	detectable	undetectable	undetectable	undetectable	undetectable	undetectable	undetectable	undetectable	undetectable
		GrpB-53	detectable	undetectable	undetectable	undetectable	0.0012	undetectable	detectable	undetectable	undetectable
		GrpB-54	detectable	undetectable	undetectable	undetectable	undetectable	undetectable	undetectable	undetectable	undetectable
		GrpB-55	0.0004	undetectable	undetectable	undetectable	undetectable	undetectable	undetectable	undetectable	undetectable
	4e8 IU/kg	GrpC-51	0.0049	0.0007	detectable	detectable	undetectable	undetectable	undetectable	undetectable	undetectable
		GrpC-52	0.0041	0.0021	0.0006	detectable	detectable	undetectable	undetectable	undetectable	undetectable
		GrpC-53	0.0017	detectable	detectable	detectable	undetectable	undetectable	undetectable	undetectable	undetectable
		GrpC-54	0.0017	0.0006	detectable	detectable	undetectable	undetectable	undetectable	detectable	undetectable
		GrpC-55	0.0029	detectable	0.0009	detectable	undetectable	undetectable	undetectable	undetectable	undetectable
Day 10	PBS	N/A	N/A	N/A	N/A	N/A	N/A	N/A	N/A	N/A	N/A
	4e7 IU/kg	GrpB-56	0.0047	0.0007	0.0017	detectable	0.0005	detectable	detectable	detectable	detectable
		GrpB-57	0.0088	not reportable	0.0013	detectable	0.0007	undetectable	undetectable	undetectable	undetectable
		GrpB-58	0.0053	0.0025	0.0035	detectable	0.0019	detectable	detectable	detectable	detectable
		GrpB-59	0.0086	0.0019	0.0035	N/S	0.0018	undetectable	detectable	undetectable	detectable
		GrpB-60	0.005	0.0012	0.001	undetectable	0.0006	undetectable	detectable	undetectable	undetectable
	4e8 IU/kg	GrpC-56	0.0532	0.0023	0.0123	0.1163	0.0108	detectable	detectable	detectable	detectable
		GrpC-57	0.0685	0.0036	0.0195	0.1242	0.0127	0.0005	undetectable	0.0004	0.0015
		GrpC-58	0.1088	0.0075	0.0342	0.1761	0.0191	0.0007	detectable	0.0007	0.0022
		GrpC-59	0.0091	0.0044	0.0200	0.1055	0.0131	0.0006	detectable	detectable	0.0018
		GrpC-60	0.1248	0.0065	0.0193	0.0806	0.0211	detectable	detectable	detectable	0.0013
Day 14	PBS	N/A	N/A	N/A	N/A	N/A	N/A	N/A	N/A	N/A	N/A
	4e7 IU/kg	GrpB-61	0.0680	0.0044	0.0329	0.1166	0.0210	0.0032	0.0050	detectable	0.0039
		GrpB-62	0.0444	0.0047	0.0649	0.1762	0.0365	0.0113	0.0102	0.0009	0.0049
		GrpB-63	0.1124	0.0143	0.0462	0.1337	0.0340	0.0115	0.0117	0.0004	0.0053
		GrpB-64	0.0637	0.0051	0.0493	0.1478	0.0215	0.0029	0.0039	0.0004	0.0046
		GrpB-65	0.0672	0.0071	0.0431	0.1652	0.0168	0.0050	0.0072	detectable	0.0020
	4e8 IU/kg	GrpC-61	0.1363	0.0187	0.1150	0.5740	0.0575	0.0068	0.0050	0.0004	0.0038
		GrpC-62	0.2415	0.0492	0.1703	0.4324	0.1123	0.0336	0.0102	0.0020	0.0101
		GrpC-63	0.2398	0.0268	0.1563	0.2526	0.0672	0.0154	0.0117	0.0004	0.0045
		GrpC-64	0.0675	0.0135	0.0815	0.4793	0.0329	0.0175	0.0039	0.0010	0.0035
		GrpC-65	0.1832	0.0167	0.1735	0.4079	0.0840	0.0238	0.0072	0.0004	0.0067

Note: LOD for this assay was ≥3 copies per reaction. LLOQ for the assay was ≥15 copies per reaction.

While the observed PK, biodistribution, and anti-tumor activity will be important in defining dosing considerations and clinical trial design for human studies, these xenograft models are not informative on the safety of the vector treatment. Several factors must be considered when interpreting non-clinical *in vivo* data and translating these findings to clinical testing. Specifically, limitations inherent to xenograft models, such as cross-species immune incompatibility and non-physiological target cell expansion, confound safety interpretation, while still allowing meaningful assessment of general PK and PD parameters, including vector distribution, clearance kinetics, and functional activity. Immunocompetent animal models may be better suited to evaluate immune-mediated clearance, vector immunogenicity, and dosing dynamics; however, current vectors and CAR constructs are not cross reactive with murine systems, and mouse T cells are weakly permissive to lentiviral transduction, limiting the utility of standard models.[58] NHP studies may provide more informative safety and PK/PD data (i.e., understanding off-target transduction). However, differences in CAR transgenes (e.g., surrogate), restriction factors, and immune responses between primate species and humans must be considered.[59–61] This study focused on $CD8^+$ T cell targeting, but similar fusosome strategies could be adapted for other T cell subsets, such as $CD4^+$ or $CD3^+$ T cells.[26,27] While targeting CD3 offers access to CD4 and CD8 T cell subsets, it raises potential safety concerns due to the potential T cell activation upon engagement.[62] The PK and PD profiles of such vectors may differ significantly and will require independent evaluation. Due to the cross-species reactivity of the targeting moiety in the fusosome, future preclinical pharmacology studies in NHP will be essential to evaluate safety, optimize dosing, and advance to clinical translation.

In summary, this work demonstrates that CD8-targeted fusosomes can mediate the efficient, potent, and dose-dependent generation of functional CAR-T cells *in vivo*, resulting in control of malignant cells. Collectively, these results support further development of this platform in patients.

MATERIALS AND METHODS

Primary and tumor cell lines

Sana Biotechnology, Inc. custom-generated the NALM6 mWasabi:ffluc LV tumor cell line using the NALM6 cells received from ATCC. The NALM6 cell line was maintained in Roswell Park Memorial Institute 1640 (RPMI-1640) medium (StemCell Technologies) supplemented with FBS (Gibco). Tumor cells were split at least three times prior to *in vivo* administration to ensure proper growth kinetics. Human PBMCs were purchased from StemCell Technologies. All cell lines were IDEXX tested to ensure they were free of mycoplasma and pathogen contamination prior to *in vivo* administration. In short, cells were thawed in a 37°C water bath and washed with RPMI prior to seeding (NALM6) or *in vivo* administration. All procedures were conducted in a BSL2 laboratory, and proper precautions were taken to prevent exposure to laboratory personnel.

Generation of fusosome

Fusosomes were produced using transient transfection of a clonal producer cell line derived in-house from the HEK293 cells with PEI-pro and transfer plasmids encoding a FMC63 CD19 CAR transgene alongside packaging plasmids pMDLg/pRRE (Gag/Pol) and pRSV-Rev, in addition to paramyxovirus F and G envelope plasmids. The G envelope plasmid was re-targeted from its cognate receptor to an anti-CD8 binder. Post-transfection, the cultures were supplemented with sodium butyrate, and the LVV-containing media were harvested and passed through a bottle-top filter unit and transferred to conical tubes. For this purpose, the LVV-containing media were underlaid with a sucrose cushion and centrifuged overnight. Viral productions were generated in 50-L batches to produce sufficient material for dosing mice. To increase the concentration of the LVV, samples are concentrated up to 1,000-fold. Prior to the release of the LVV for *in vivo* administration, a series of analytical tests were run to confirm the functional and physical titer of the production, as well as endotoxin levels.

Ethical statements

This study was conducted in accordance with the Animal Welfare Act regulations (Code of Federal Regulations, Title 9), the Public Health Service Policy on Humane Care and Use of Laboratory Animals from the Office of Laboratory Animal Welfare, the Charles River Accelerator and Development Labs (CRADL) Institutional Animal Care and Use Committee (IACUC), and the Guide for the Care and Use of Laboratory Animals from the National Research Council.

Male and Female NOD.Cg-Prkdcscid Il2rgtm1Wjl/SzJ NSG mice of 6–8 weeks of age were purchased from Jackson Laboratories and were housed in rooms at 68°F–79°F, 30%–70% humidity, and an automated 12-h light/dark cycle. Food and water were provided ad libitum. All animals in a group of five mice per cage were housed in an innovative filter caging system with disposable caging materials that were double-bagged and irradiated for disposal.

Animal identifications were recorded and tracked in SoftMouse Internet Colony Management Software (Iseehear Inc. Life Sciences). Animals were inspected, weighed, and acclimated for at least 72 h before the study's initiation. Trained animal care staff visually inspected all animals daily, and veterinary aid was provided if needed, at any time during the week. Animals were euthanized if they lost more than 20% of their weight, exhibited internal hemorrhage/cyanosis, showed signs of shortness of breath/labored breathing, or displayed general morbidity. A body condition score (BCS) of 2 or less was considered a basis for euthanasia. If pain or distress signs were observed, a wet food/gel diet, subcutaneous (SC) fluid injection (500 μL), or thermal support was used to comfort mice.

Animal models and tissue collection

Female and/or male NSG mice were allowed to acclimate for at least 72 h before study initiation. All animals were weighed for a baseline measurement before any treatment to monitor treatment-related

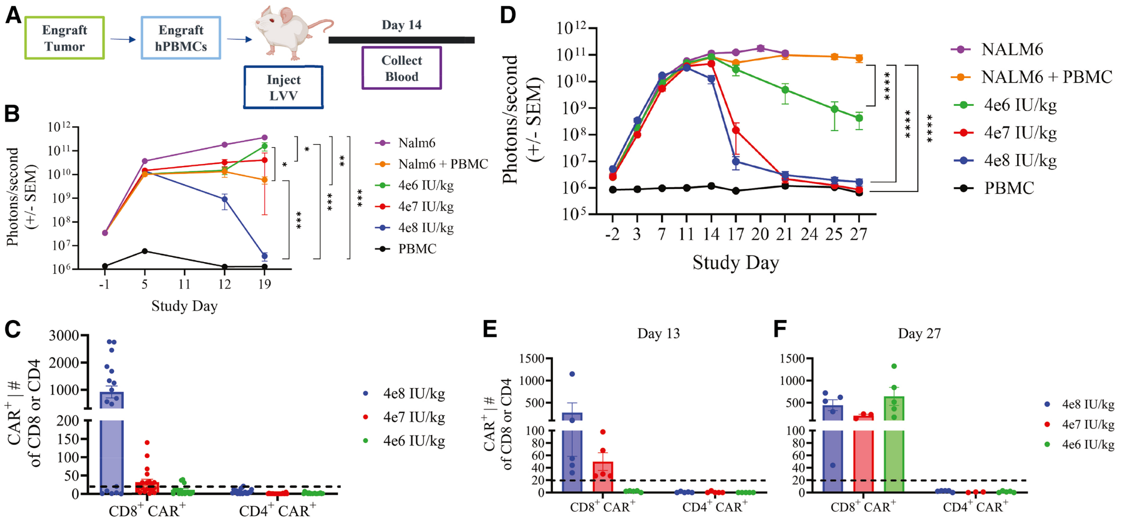

Figure 4. Dose-dependent tumor suppression and CAR-T cell detection

(A) Study design schematic of male and female NSG mice (n = 10/sex/group) engrafted with CD19+ NALM6 tumor cells on day 3, human PBMCs on day 1, and LVV on day 0. Animals were dosed with 4e8 IU/kg of LVV (1e7 IU/mouse), 4e7 IU/kg of LVV (1e6 IU/mouse), or 4e6 IU/kg of LVV (1e5 IU/mouse). Blood was collected 14 days post-fusosome injection. (B) Average photons per second (±SEM) values (tumor burden) of the first PBMC donor graphed over time in PBMC (black), NALM6 (purple), PBMC and NALM6 (orange), 4e6 IU/kg (green), 4e7 IU/kg (red), and 4e8 IU/kg (blue) of LVV. (C) Number of CD8+CAR+ or CD4+CAR+ cells per 100 μL of blood from donor 1 measured by flow cytometry on day 14 post-administration with 4e6 IU/kg (green), 4e7 IU/kg (red), and 4e8 IU/kg (blue) of LVV. The dotted line represents the limit of detection of the assay (>20 events). (D) Average photons per second (±SEM) values graphed over time for the second PBMC donor showing PBMC (black), NALM6 (purple), PBMC and NALM6 (orange), 4e6 IU/kg (green), 4e7 IU/kg (red), and 4e8 IU/kg (blue) of LVV. (E) Number of CD8+CAR+ or CD4+CAR+ cells per 100 μL of blood from donor 2 measured by flow cytometry on day 13 post-administration in 4e6 IU/kg (green), 4e7 IU/kg (red), and 4e8 IU/kg (blue)-dose-treated mice. The dotted line represents the limit of detection of the assay (>20 events). (F) Number of CD8+CAR+ or CD4+CAR+ cells per 100 μL of blood from donor 2 measured by flow cytometry on day 27 post-administration in 4e6 IU/kg (green), 4e7 IU/kg (red), and 4e8 IU/kg (blue)-dose-treated mice. The dotted line represents the limit of detection of the assay (>20 events). Statistical significance was measured using a linear mixed effects model with a pairwise comparison with a Wilcoxon rank-sum test or a one-way ANOVA with a Bonferroni multiple comparison (*$p < 0.05$, **$p < 0.01$, ***$p < 0.005$, ****$p < 0.001$). Error bars represent standard error of the mean (SEM).

body weight changes. NSG mice being evaluated for CD19+-targeted tumor killing received CD19+ NALM6 tumor cells on study day 3. On study day 1, the mice were administered human PBMCs intravenously or did not receive PBMCs. Animals were then dosed intravenously with fusosome at the doses indicated or PBS on study day 0. Fusosome dosing was normalized to individual animal body weight (IU/kg; ranging from 17 to 25 grams), with animals weighed immediately prior to administration to ensure accurate dose delivery.

To evaluate fusosome clearance and biodistribution, animals were either sacrificed at each time point, or plasma samples were collected using a microsampling technique. A series of blood samples were collected from each mouse at 10, 20, 30, 40, and 50 min and 1 and 2 h post-dose. Alternatively, blood and tissues (lung, liver, spleen) were collected at 10, 20, 40, 60, and 90 min; 2, 3, 6, 24, and 72 h; and 1 week post-dose. Blood and tissues from vehicle control animals were collected at the initial and final times post-dose. Additionally, tissue samples (spleen, bone marrow, lung, whole blood, liver, gonads, kidneys, brain, and heart) were collected 7, 10, and 14 days post-fusosome administration.

For microsampling, the tail was snipped with a disposable scalpel, and 10 μL of blood was collected into a microvette and slowly ejected into a 1.5 mL microcentrifuge tube containing 190 μL of ice-cold, liquid micro-sampling buffer. After mixing, plasma was isolated by centrifugation, aliquoted, and frozen on dry ice. Samples were stored in −80°C freezer until analyzed.

Cardiac blood was collected by cardiac puncture and used for flow cytometry analysis. After 100 μL blood collected from the cardiac puncture in a K2EDTA tube was separated for flow cytometry, the remaining blood was lysed using the Macherey-Nagel NuceloSpin Tissue Kit (Macherey-Nagel, Cat# 740952) for VCN analysis. Proteinase K solution 25 μL was directly added to 200 μL of blood, followed by the addition of 200 μL of Buffer B3. After vortexing, the sample was incubated at room temperature for 5 min, aliquoted, and stored on dry ice until transferred to −80°C for storage.

Tissues were harvested after euthanasia. The spleen, liver, and lung were aliquoted and snap-frozen in liquid nitrogen for GQA analysis. The brain, spleen, liver, lung, heart, ovary, and kidney were aliquoted and snap-frozen in liquid nitrogen for VCN analysis. The spleen, liver, and lung were kept on wet ice until processed for flow cytometry. Bone marrow was expelled from the bone and centrifuged, and the cells were aliquoted and frozen on dry ice for VCN analysis. Bone marrow cells collected for flow cytometry were kept on wet ice until

analyzed. Tissues collected for GQA and VCN analysis were stored at −80°C until analyzed.

Lenti viral genome quantification

Viral genome quantitation (GQA) was performed based on one-step RT-ddPCR using QX200 Bio-Rad platform for the analysis. In this assay, RNA extraction lysis buffer (Thermo Fisher Scientific, Cat# A27828) was spiked with an external RNA normalizer (mKate2) prior to RNA extraction from plasma. Plasma samples were treated with DNAse, and reverse transcription was performed using sets of fluorescent primers/probes for genomic viral RNA and mKate2. Results were normalized to mKate2 values. Quantifiable values were reported if greater than or equal to 60–100,000 GQA copies per reaction with a CV less than 40%. Values were detectable if they were within 15 and 60 GQA copies per reaction and undetectable under 15 copies per reaction. The RT-ddPCR assay for GQA consists of an oligonucleotide mix of primers and probes, which are listed in Table S1.

Genome integration

Genomic DNA (gDNA) was extracted from each test sample using a Qiagen kit (Catalog # 51306). Extracted gDNA concentrations were measured with a Nanodrop instrument and PicoGreen kit (Thermo Fisher Scientific, Catalog #P7589). After extraction, each sample was diluted to 29 ng/μL, and a total of 290 ng DNA was tested in the final ddPCR reaction. A volume of 12.5 μL of the diluted samples was directly used for ddPCR template. For the whole blood samples, the DNA was extracted following the protocol for the NucleoSpin 96 Tissue Core Kit (a 96-well kit for DNA preparation from cells and tissue, Macherey-Nagel Catalog # 740454.4). The extracted DNA was quantified by NanoDrop and PicoGreen. If the concentration of all extracted DNA was less than 29 ng/μL, 12.5 μL of undiluted DNA was used in the reaction.

VCN was quantified in whole blood, lung, liver, spleen, brain, heart, kidney, femur, tibia, and gonads (ovary or testis). Samples were analyzed using a ddPCR-platform-based method that was developed to detect and quantify CD19-CAR-specific sequences in mouse matrices. A set of fluorescent primers was used to detect the reference gene *ARX* (Pol-A gene). The duplex assay measured copies of CD19-CAR and *ARX* in each ddPCR well/sample (Table S2). VCN per diploid genome was calculated as the ratio of copies (CD19-CAR: *ARX*) × 2 for females and × 1 for males.

Two sets of high-quality control (HQC), low-quality control (LQC), negative control (NC), and no-template control (NTC) were applied to every sample testing plate and used to evaluate the assays' acceptance. Fifty percent of QC samples at each level needed to be within acceptance criteria for %RE (relative error) and %CV (coefficient of variation) to pass acceptance.

Samples were quantifiable and reported if they contained more than 15 copies/reaction of target copies per measurement (qualified LLOQ level) and a %CV $\leq$ 40%. All numbers lower than 15 copies per reaction were reported as undetectable.

Flow cytometry

Engraftment of human PBMCs and expression of anti-CD19 CAR on $CD8^+$ T cells was determined by flow cytometry in blood, liver, spleen, and bone marrow. Red blood cells in the blood and single-cell suspensions of tissues were removed using RBC lysis buffer (Thermo Fisher Scientific). Prewashed cells were blocked using 1:50 diluted Human TruStain FcX blocking solution (BioLegend) and incubated with live-dead stain, followed by antibodies for 30 min on ice. The reagents used for staining the cells included APC-H7 Fixable Viability Dye (Thermo Fisher Scientific); BV650 anti-human CD3, BV711 anti-human CD19, and BV421 anti-human CD45 (BioLegend); and PE-CF594 anti-human CD4, AF647 anti-human CD8, and BUV496 anti-mouse CD45 (BD Biosciences). PE anti-FMC63 ScFv antibody was purchased from Acro Biosystems. After washing, samples were fixed in a fixation buffer (BD Biosciences). Samples were acquired on CytoFLEX LX (Beckman Coulter) and analyzed with FlowJo software. The limit of detection for flow cytometry was greater than 20 events.

Bioluminescent imaging

Bioluminescent imaging (BLI) on a Lago-X spectral imager (Spectral Instruments Imaging, Arizona) of the NALM6 mWasabi:ffluc cells was performed weekly to monitor tumor burden. NALM6 mWasabi:ffLuc is a B cell precursor leukemia cell line engineered to express luciferase. When injected with luciferin, the animal emits a signal that is classified as tumor burden. Tumor burden is quantified by calculating the total flux (photons/second) emitted from regions of interest (ROIs) around individual mice.

Mice were injected intraperitoneally with 150 μL of luciferin (PerkinElmer) at a concentration of 150 mg/mL. They were anesthetized with isoflurane while imaging. Bioluminescence was acquired on the Spectral Lago-X imaging system (Spectral Instruments Imaging) and analyzed using the Aura imaging and analysis software (Spectral Instruments Imaging).

PK and statistical analysis

An external contract research organization (Syneos Health Clinique Inc.) calculated PK parameters for PK studies. AUC_{0-t}, C_{max}, C_{last}, T_{max}, T_{last}, and $T_{1/2}$ were calculated from the mean viral genome concentration data using standard noncompartmental methods consistent with the IV bolus route of administration. Additionally, partial AUCs from time 0 to 30 min (AUC_{0-30}) and 30 to 90 min (AUC_{30-90}) were calculated from plasma concentration data, and partial AUCs from time 0 to 90 min (AUC_{0-90}) and 0 to 360 min (AUC_{0-360}) were calculated from liver, lung, and spleen concentration data. AUCs were calculated using the linear trapezoidal method when concentrations were increasing and the logarithmic trapezoidal method when concentrations were decreasing (i.e., Linear Up Log Down). The slope of the terminal elimination phase of the mean concentrations versus time curves of each treatment group was

estimated and used to calculate the $T_{1/2}$ and determined by log-linear regression. R_{sq} adjusted the goodness of fit statistic for the terminal elimination phase.

All other statistical analyses were conducted in Microsoft Excel or GraphPad Prism. Flow cytometry analyses were assessed for statistical significance with an unpaired nonparametric Mann-Whitney test. Statistical analysis of tumor suppression was performed by calculating the AUC for each mouse, which was then evaluated for significance using a one-way ANOVA with Bonferroni's multiple comparison test, a linear mixed effects model, and a pairwise comparison with a Wilcoxon Rank Sum Test or one-way ANOVA with a Bonferroni correction. Due to the nature of the VCN results yielding detectable values, no significance testing was performed to avoid biasing the data. Overall, a p value less than 0.05 was considered significant ($^{*}p < 0.05$, $^{**}p < 0.01$, $^{***}p < 0.005$, $^{****}p < 0.001$).

DATA AND CODE AVAILABILITY

The data that support the findings of this study are available from the corresponding author upon reasonable request.

ACKNOWLEDGMENTS

We gratefully acknowledge the valuable contributions of both past and present Sana Biotechnology, Inc. employees, whose dedication, expertise, and efforts have supported this work. We also want to acknowledge BioRender for the mouse image used in the main text figures. The image was created in BioRender. Duback, T. (2026) https://BioRender.com/bw6n07s.

AUTHOR CONTRIBUTIONS

V.D. was responsible for conceptualization, formal analysis, investigation, methodology, project administration, visualization, writing the original draft, and reviewing and editing of the manuscript. S.S.K. was responsible for conceptualization, investigation, methodology, and project administration. V.V., K.P., A,S., J.K., A.G., J.G., B.D., K.H., G.Z, and S.J., were responsible for investigation. S.J. was also responsible for project administration. B.V., K.T., J.S., T.F., and K.G.E. were also responsible for methodology and conceptualization. B.V. and K.G.E. were responsible for supervision. K.G.E. was responsible for validation. J.G., B.D., A.K., K.T., and K.G.E. were also responsible for reviewing and editing the manuscript.

DECLARATION OF INTERESTS

V.D., K.P., A.A., J.G., B.D., G.Z., S.J., K.T., and K.G.E. are current employees and shareholders of Sana Biotechnology, Inc. T.F. is a consultant and shareholder of Sana Biotechnology, Inc. S.S.K., V.V., A.S., J.K., K.H., N.V.H., V.C., O.L., J.M., A.O., B.V., and J.S. are former employees and potential shareholders of Sana Biotechnology, Inc.

SUPPLEMENTAL INFORMATION

Supplemental information can be found online at https://doi.org/10.1016/j.omta.2026.201803.

REFERENCES

1. Mitra, A., Barua, A., Huang, L., Ganguly, S., Feng, Q., and He, B. (2023). From bench to bedside: the history and progress of CAR T cell therapy. Front. Immunol. *14*, 1188049. https://doi.org/10.3389/fimmu.2023.1188049.
2. De Marco, R.C., Monzo, H.J., and Ojala, P.M. (2023). CAR T Cell Therapy: A Versatile Living Drug. Int. J. Mol. Sci. *24*, 6300. https://doi.org/10.3390/ijms24076300.
3. June, C.H., O'connor, R.S., Kawalekar, O.U., Ghassemi, S., and Milone, M.C. (2018). CAR T cell immunotherapy for human cancer. Science *359*, 1361–1365. https://doi.org/10.1126/science.aar6711.
4. Almåsbak, H., Aarvak, T., and Vemuri, M.C. (2016). CAR T Cell Therapy: A Game Changer in Cancer Treatment. J. Immunol. Res. *2016*, 5474602. https://doi.org/10.1155/2016/5474602.
5. Shah, N.N., and Fry, T.J. (2019). Mechanisms of resistance to CAR T cell therapy. Nat. Rev. Clin. Oncol. *16*, 372–385. https://doi.org/10.1038/s41571-019-0184-6.
6. Ayala Ceja, M., Khericha, M., Harris, C.M., Puig-Saus, C., and Chen, Y.Y. (2024). CAR-T cell manufacturing: Major process parameters and next-generation strategies. J. Exp. Med. *221*, e20230903. https://doi.org/10.1084/jem.20230903.
7. Sterner, R.C., and Sterner, R.M. (2021). CAR-T cell therapy: current limitations and potential strategies. Blood Cancer J. *11*, 69. https://doi.org/10.1038/s41408-021-00459-7.
8. Zheng, R., Zhu, X., and Xiao, Y. (2024). Advances in CAR-T-cell therapy in T-cell malignancies. J. Hematol. Oncol. *17*, 49. https://doi.org/10.1186/s13045-024-01568-z.
9. Sasu, B.J., Lauron, E.J., Schulz, T., Cheng, H.-Y., and Sommer, C. (2024). Allogeneic CAR T Cell Therapy for Cancer. Ann. Rev. Cancer Biol. *8*, 227–243. https://doi.org/10.1146/annurev-cancerbio-062822.
10. Wang, Z., Wu, Z., Liu, Y., and Han, W. (2017). New development in CAR-T cell therapy. J. Hematol. Oncol. *10*, 53. https://doi.org/10.1186/s13045-017-0423-1.
11. Xin, T., Cheng, L., Zhou, C., Zhao, Y., Hu, Z., and Wu, X. (2022). In-Vivo Induced CAR-T Cell for the Potential Breakthrough to Overcome the Barriers of Current CAR-T Cell Therapy. Front. Oncol. *12*, 809754. https://doi.org/10.3389/fonc.2022.809754.
12. Jin, K.T., Chen, B., Liu, Y.Y., Lan, H.U.R., and Yan, J.P. (2021). Monoclonal antibodies and chimeric antigen receptor (CAR) T cells in the treatment of colorectal cancer. Cancer Cell Int. *21*, 83. https://doi.org/10.1186/s12935-021-01763-9.
13. Short, L., Holt, R.A., Cullis, P.R., and Evgin, L. (2024). Direct in vivo CAR T cell engineering. Trends Pharmacol. Sci. *45*, 406–418. https://doi.org/10.1016/j.tips.2024.03.004.
14. Wang, R., Yu, J., Caligiuri, M.A., and Ma, S. (2026). Optimizing In Vivo CAR-T Cell Engineering for Cancer Immunotherapy. Cancer Res. *86*, 1823–1835. https://doi.org/10.1158/0008-5472.CAN-25-3748.
15. Lundstrom, K. (2023). Viral Vectors in Gene Therapy: Where Do We Stand in 2023? Viruses *15*, 698. https://doi.org/10.3390/v15030698.
16. Bot, A., Scharenberg, A., Friedman, K., Guey, L., Hofmeister, R., Andorko, J.I., Klichinsky, M., Neumann, F., Shah, J.V., Swayer, A.J., et al. (2026). In vivo chimeric antigen receptor (CAR)-T cell therapy. Nat. Rev. Drug Discov. *25*, 116–137. https://doi.org/10.1038/s41573-025-01291-5.
17. Wang, C., Pan, C., Yong, H., Wang, F., Bo, T., Zhao, Y., Ma, B., He, W., and Li, M. (2023). Emerging non-viral vectors for gene delivery. J. Nanobiotechnol. *21*, 272. https://doi.org/10.1186/s12951-023-02044-5.
18. Tsuchida, C.A., Wasko, K.M., Hamilton, J.R., and Doudna, J.A. (2024). Targeted nonviral delivery of genome editors in vivo. Proc. Natl. Acad. Sci. USA *121*, e2307796121. https://doi.org/10.1073/pnas.2307796121.
19. Wang, Q., Xiao, Z.X., Zheng, X., Wang, G., Yang, L., Shi, L., Xiang, N., Wang, X., Zha, G.-F., Schett, G., and Chen, Z. (2025). In Vivo CD19 CAR T-Cell Therapy for Refractory Systemic Lupus Erythematosus. N. Engl. J. Med. *393*, 1542–1544. https://doi.org/10.1056/nejmc2509522.
20. Rasbach, A., Abel, T., Münch, R.C., Boller, K., Schneider-Schaulies, J., and Buchholz, C.J. (2013). The Receptor Attachment Function of Measles Virus Hemagglutinin Can Be Replaced with an Autonomous Protein That Binds Her2/neu While Maintaining Its Fusion-Helper Function. J. Virol. *87*, 6246–6256. https://doi.org/10.1128/jvi.03298-12.
21. Bender, R.R., Muth, A., Schneider, I.C., Friedel, T., Hartmann, J., Plückthun, A., Maisner, A., and Buchholz, C.J. (2016). Receptor-Targeted Nipah Virus Glycoproteins Improve Cell-Type Selective Gene Delivery and Reveal a Preference for Membrane-Proximal Cell Attachment. PLoS Pathog. *12*, e1005641. https://doi.org/10.1371/journal.ppat.1005641.
22. Duprex, W.P., and Dutch, R.E. (2023). Paramyxoviruses: Pathogenesis, Vaccines, Antivirals, and Prototypes for Pandemic Preparedness. J. Infect. Dis. *228*, S390–S397. https://doi.org/10.1093/infdis/jiad123.

23. Nicolai, C.J., Parker, M.H., Qin, J., Tang, W., Ulrich-Lewis, J.T., Gottschalk, R.J., Cooper, S.E., Hernandez Lopez, S.A., Parrilla, D., Mangio, R.S., et al. (2024). In vivo CAR T-cell generation in non-human primates using lentiviral vectors displaying a multi-domain fusion ligand. Blood *144*, 977–987. https://doi.org/10.1182/blood.2024024523.

24. Michels, K.R., Sheih, A., Hernandez, S.A., Brandes, A.H., Parrilla, D., Irwin, B., Perez, A.M., Ting, H.A., Nicolai, C.J., Gervascio, T., et al. (2023). Preclinical proof of concept for VivoVec, a lentiviral-based platform for in vivo CAR T-cell engineering. J. Immunother. Cancer *11*, e006292. https://doi.org/10.1136/jitc-2022-006292.

25. Agarwal, S., Weidner, T., Thalheimer, F.B., and Buchholz, C.J. (2019). In vivo generated human CAR T cells eradicate tumor cells. Oncoimmunology *8*, e1671761. https://doi.org/10.1080/2162402X.2019.1671761.

26. Agarwal, S., Hanauer, J.D.S., Frank, A.M., Riechert, V., Thalheimer, F.B., and Buchholz, C.J. (2020). In Vivo Generation of CAR T Cells Selectively in Human CD4+ Lymphocytes. Mol. Ther. *28*, 1783–1794. https://doi.org/10.1016/j.ymthe.2020.05.005.

27. Frank, A.M., Braun, A.H., Scheib, L., Agarwal, S., Schneider, I.C., Fusil, F., Perian, S., Sahin, U., Thalheimer, F.B., Verhoeyen, E., and Buchholz, C.J. (2020). Combining T-cell-specific activation and in vivo gene delivery through CD3-targeted lentiviral vectors. Blood Adv. *4*, 5702–5715. https://doi.org/10.1182/bloodadvances.2020002229.

28. Mullard, A. (2024). In vivo CAR T cells move into clinical trials. Nat. Rev. Drug Discov. *23*, 727–730. https://doi.org/10.1038/d41573-024-00150-z.

29. An, N., Wang, D., Zhang, P., Zhang, J., Parone, P., Hu, J., Bao, Y., Xu, L., Ruan, H., Wan, Y., et al. (2026). In vivo generation of anti-BCMA CAR-T cells in relapsed or refractory multiple myeloma: a phase 1 study. Nat. Med. *32*, 1257–1266. https://doi.org/10.1038/s41591-026-04244-6.

30. Xu, J., Liu, L., Parone, P., Xie, W., Sun, C., Chen, Z., Zhang, J., Li, C., Hu, Y., and Mei, H. (2025). In-vivo B-cell maturation antigen CAR T-cell therapy for relapsed or refractory multiple myeloma. Lancet *406*, 228–231. https://doi.org/10.1056/nejmp2502567.

31. Dambra, R., Matter, A., Graca, K., Akhand, S.S., Mehta, S., Bell-Cohn, A., Swenson, J.M., Abid, S., Xin, D., Lewis, C., et al. (2023). Nonclinical pharmacokinetics and biodistribution of VSV-GP using methods to decouple input drug disposition and viral replication. Mol. Ther. Methods Clin. Dev. *28*, 190–207. https://doi.org/10.1016/j.omtm.2022.12.013.

32. Kavita, U., Sun, K., Braun, M., Lembke, W., Mody, H., Kamerud, J., Yang, T.Y., Braun, I.V., Fang, X., Gao, W., et al. (2023). PK/PD and Bioanalytical Considerations of AAV-Based Gene Therapies: an IQ Consortium Industry Position Paper. AAPS J. *25*, 78. https://doi.org/10.1208/s12248-023-00842-1.

33. Reichenbach, P., Giordano Attianese, G.M.P., Ouchen, K., Cribioli, E., Triboulet, M., Ash, S., Saillard, M., Vuillefroy de Silly, R., Coukos, G., and Irving, M. (2023). A lentiviral vector for the production of T cells with an inducible transgene and a constitutively expressed tumour-targeting receptor. Nat. Biomed. Eng. *7*, 1063–1080. https://doi.org/10.1038/s41551-023-01013-5.

34. Krug, A., Saidane, A., Martinello, C., Fusil, F., Michels, A., Buchholz, C.J., Ricci, J.E., and Verhoeyen, E. (2024). In vivo CAR T cell therapy against angioimmunoblastic T cell lymphoma. J. Exp. Clin. Cancer Res. *43*, 262. https://doi.org/10.1186/s13046-024-03179-5.

35. Liu, S., Chowdhury, E.A., Xu, V., Jerez, A., Mahmood, L., Ly, B.Q., Le, H.K., Nguyen, A., Rajwade, A., Meno-Tetang, G., and Shah, D.K. (2024). Whole-Body Disposition and Physiologically Based Pharmacokinetic Modeling of Adeno-Associated Viruses and the Transgene Product. J. Pharm. Sci. *113*, 141–157. https://doi.org/10.1016/j.xphs.2023.10.005.

36. Batty, P., and Lillicrap, D. (2024). Adeno-associated viral vector integration: implications for long-term efficacy and safety. J. Thromb. Haemostasis *22*, 2945–2960. https://doi.org/10.1016/j.jtha.2024.07.012.

37. Van Gestel, M.A., Boender, A.J., De Vrind, V.A.J., Garner, K.M., Luijendijk, M.C.M., and Adan, R.A.H. (2014). Recombinant adeno-associated virus: Efficient transduction of the rat VMH and clearance from blood. PLoS One *9*, e97639. https://doi.org/10.1371/journal.pone.0097639.

38. Kaiser, C.W., Rouchka, E.C., and Smith, M.L. (2026). Adaptation of lentiviral vectors for viral gene therapy and their impact on host cell biology. J. Transl. Med. *24*, 272. https://doi.org/10.1186/s12967-025-07626-5.

39. Nicholls, B.R.M., Johnson, D., Kulkarni, A., Raposo, R.A.S., Mitrophanous, K.A., Coussios, C.C., and Carlisle, R.C. (2026). Normothermic perfusion of human livers for profiling lentiviral vector pharmacokinetics and transduction. Mol. Ther. Adv. *34*, 201660. https://doi.org/10.1016/j.omta.2025.201660.

40. Pan, D., Gunther, R., Duan, W., Wendell, S., Kaemmerer, W., Kafri, T., Verma, I.M., and Whitley, C.B. (2002). Biodistribution and toxicity studies of VSVG-pseudotyped lentiviral vector after intravenous administration in mice with the observation of in vivo transduction of bone marrow. Mol. Ther. *6*, 19–29. https://doi.org/10.1006/mthe.2002.0630.

41. Peng, K.-W., Pham, L., Ye, H., Zufferey, R., Trono, D., Cosset, F.-L., and Russell, S.J. (2001). Organ distribution of gene expression after intravenous infusion of targeted and untargeted lentiviral vectors. Gene Ther. *8*, 1456–1463. https://doi.org/10.1038/sj.gt.3301552.

42. Andorko, J.I., Russell, R.M., Schnepp, B.C., Grubaugh, D., Mullen, K.F., Wakabayashi, A., Carrington, L.J., O'Malley, T., Kuri-Cervantes, L., Culp, T.D., and Johnson, P.R. (2025). Targeted in vivo delivery of genetic medicines utilizing an engineered lentiviral vector platform results in CAR T and NK cell generation. Mol. Ther. *33*, 4937–4952. https://doi.org/10.1016/j.ymthe.2025.06.036.

43. Reuter, J.D., Fang, X., Ly, C.S., Suter, K.K., and Gibbs, D. (2012). Assessment of Hazard Risk Associated with the Intravenous Use of Viral Vectors in Rodents. Comp. Med. *62*, 361–370.

44. Clark, A., Hammon, K., Sandza, K., Torres, R., Koziol, E., Holcomb, J., Kim, B., Jayaram, K., Russell, C., Vettermann, C., and Henshaw, J. (2020). Vector Shedding and Blood Biodistribution in Patients with Severe Hemophilia a Following Administration of Valoctocogene Roxaparvovec. Blood *136*, 21. https://doi.org/10.1182/blood-2020-136459.

45. Youngman E.M., Kuri-Cervantes L., Edwards S., Miranda M., Schreiber C., Richardson J., Culp T., Johnson P.R..(2024) Pharmacokinetics and Vector Shedding in NHPs Following a Single Intravenous Infusion of a CD20-Targeted Engineered Lentiviral Vector, MOLECULAR THERAPY, 32, 126, Proceedings of the 27th Annual Meeting of the American Society of Gene & Cell Therapy; Baltimore, MD, USA. 7–11 May.

46. Morillon, Y.M., 2nd, Sabzevari, A., Schlom, J., and Greiner, J.W. (2020). The development of next-generation PBMC humanized mice for preclinical investigation of cancer immunotherapeutic agents. Anticancer Res. *40*, 5329–5341. https://doi.org/10.21873/anticanres.14540.

47. Ma, H., Pilvankar, M., Wang, J., Giragossian, C., and Popel, A.S. (2021). Quantitative Systems Pharmacology Modeling of PBMC-Humanized Mouse to Facilitate Preclinical Immuno-oncology Drug Development. ACS Pharmacol. Transl. Sci. *4*, 213–225. https://doi.org/10.1021/acsptsci.0c00178.

48. Jiang, J., and Ahuja, S. (2021). Addressing Patient to Patient Variability for Autologous CAR T Therapies. J. Pharm. Sci. *110*, 1871–1876. https://doi.org/10.1016/j.xphs.2020.12.015.

49. Hess, N.J., Turicek, D.P., Riendeau, J., Mcilwain, S.J., Contreras Guzman, E., Nadiminti, K., Hudson, A., Callander, N.S., Skala, M.C., Gumperz, J.E., et al. (2023). Inflammatory CD4/CD8 double-positive human T cells arise from reactive CD8 T cells and are sufficient to mediate GVHD pathology. Sci. Adv. *9*, eadf0567. https://doi.org/10.1126/sciadv.adf0567.

50. Duncan, B.B., Dunbar, C.E., and Ishii, K. (2022). Applying a clinical lens to animal models of CAR-T cell therapies. Mol. Ther. Methods Clin. Dev. *27*, 17–31. https://doi.org/10.1016/j.omtm.2022.08.008.

51. Chupp, D.P., Rivera, C.E., Zhou, Y., Xu, Y., Ramsey, P.S., Xu, Z., Zan, H., and Casali, P. (2024). A humanized mouse that mounts mature class-switched, hypermutated and neutralizing antibody responses. Nat. Immunol. *25*, 1489–1506. https://doi.org/10.1038/s41590-024-01880-3.

52. Ying, Z., He, T., Wang, X., Zheng, W., Lin, N., Tu, M., Xie, Y., Ping, L., Zhang, C., Liu, W., et al. (2021). Distribution of chimeric antigen receptor-modified T cells against CD19 in B-cell malignancies. BMC Cancer *21*, 198. https://doi.org/10.1186/s12885-021-07934-1.

53. Sta Maria, N.S., Khawli, L.A., Pachipulusu, V., Lin, S.W., Zheng, L., Cohrs, D., Liu, X., Hu, P., Epstein, A.L., and Jacobs, R.E. (2021). Spatio-temporal biodistribution of 89Zr-oxine labeled huLym-1-A-BB3z-CAR T-cells by PET imaging in a preclinical tumor model. Sci. Rep. *11*, 15077. https://doi.org/10.1038/s41598-021-94490-0.

54. Chavan, H., Amatya, S., Berlfein, V., Cruite, P., Chaturvedi, V., Vagin, V., van Hoeven, N., Shah, J., Fry, T.J., Gorovits, B., and Chandra, S. (2023). In Vitro and In Vivo Specificity and Biodistribution of a Novel CD8-Targeted Fusosome. Blood *142*, 3630. https://doi.org/10.1182/blood-2023-190158.

55. Milani, M., Annoni, A., Moalli, F., Liu, T., Cesana, D., Calabria, A., Bartolaccini, S., Biffi, M., Russo, F., Visigalli, I., et al. (2019). Phagocytosis-shielded lentiviral vectors improve liver gene therapy in nonhuman primates. Sci. Transl. Med. *11*, eaav7325. https://doi.org/10.1126/scitranslmed.aav7325.

56. Yaguchi, T., Kobayashi, A., Inozume, T., Morii, K., Nagumo, H., Nishio, H., Iwata, T., Ka, Y., Katano, I., Ito, R., et al. (2018). Human PBMC-transferred murine MHC class I/II-deficient NOG mice enable long-term evaluation of human immune responses. Cell. Mol. Immunol. *15*, 953–962. https://doi.org/10.1038/cmi.2017.106.

57. Ho, N., Agarwal, S., Milani, M., Cantore, A., Buchholz, C.J., and Thalheimer, F.B. (2022). In vivo generation of CAR T cells in the presence of human myeloid cells. Mol. Ther. Methods Clin. Dev. *26*, 144–156. https://doi.org/10.1016/j.omtm.2022.06.004.

58. Kerkar, S.P., Sanchez-Perez, L., Yang, S., Borman, Z.A., Muranski, P., Ji, Y., Chinnasamy, D., Kaiser, A.D.M., Hinrichs, C.S., Klebanoff, C.A., et al. (2011). Genetic engineering of murine CD8+ and CD4+ T cells for preclinical adoptive immunotherapy studies. J. Immunother. *34*, 343–352. https://doi.org/10.1097/CJI.0b013e3182187600.

59. Fadel, H.J., and Poeschla, E.M. (2011). Retroviral restriction and dependency factors in primates and carnivores. Vet. Immunol. Immunopathol. *143*, 179–189. https://doi.org/10.1016/j.vetimm.2011.06.002.

60. Harris, R.S., Hultquist, J.F., and Evans, D.T. (2012). The restriction factors of human immunodeficiency virus. J. Biol. Chem. *287*, 40875–40883. https://doi.org/10.1074/jbc.R112.416925.

61. Coroadinha, A.S. (2023). Host Cell Restriction Factors Blocking Efficient Vector Transduction: Challenges in Lentiviral and Adeno-Associated Vector Based Gene Therapies. Cells *12*, 732. https://doi.org/10.3390/cells12050732.

62. Morris, E.C., Neelapu, S.S., Giavridis, T., and Sadelain, M. (2022). Cytokine release syndrome and associated neurotoxicity in cancer immunotherapy. Nat. Rev. Immunol. *22*, 85–96. https://doi.org/10.1038/s41577-021-00547-6.

OMTA, Volume *34*

Supplemental information

Pharmacokinetic and pharmacodynamic characterization of CD8-targeted lentiviral vector for *in vivo* CD19-directed CAR-T therapy

Victoria Duback, Shalu S. Kharkwal, Vasily Vagin, Kerrie Paterson, Alicia Sabbio, Joohwan Kim, Amey Gaikwad, Jesse Green, Brian Dolinski, Kelan Hlavaty, Garrett Zipp, Shannon Joyce, Abigail Koppes, Bindu Varghese, Kyle Trudeau, Jagesh Shah, Terry Fry, and Kutlu G. Elpek

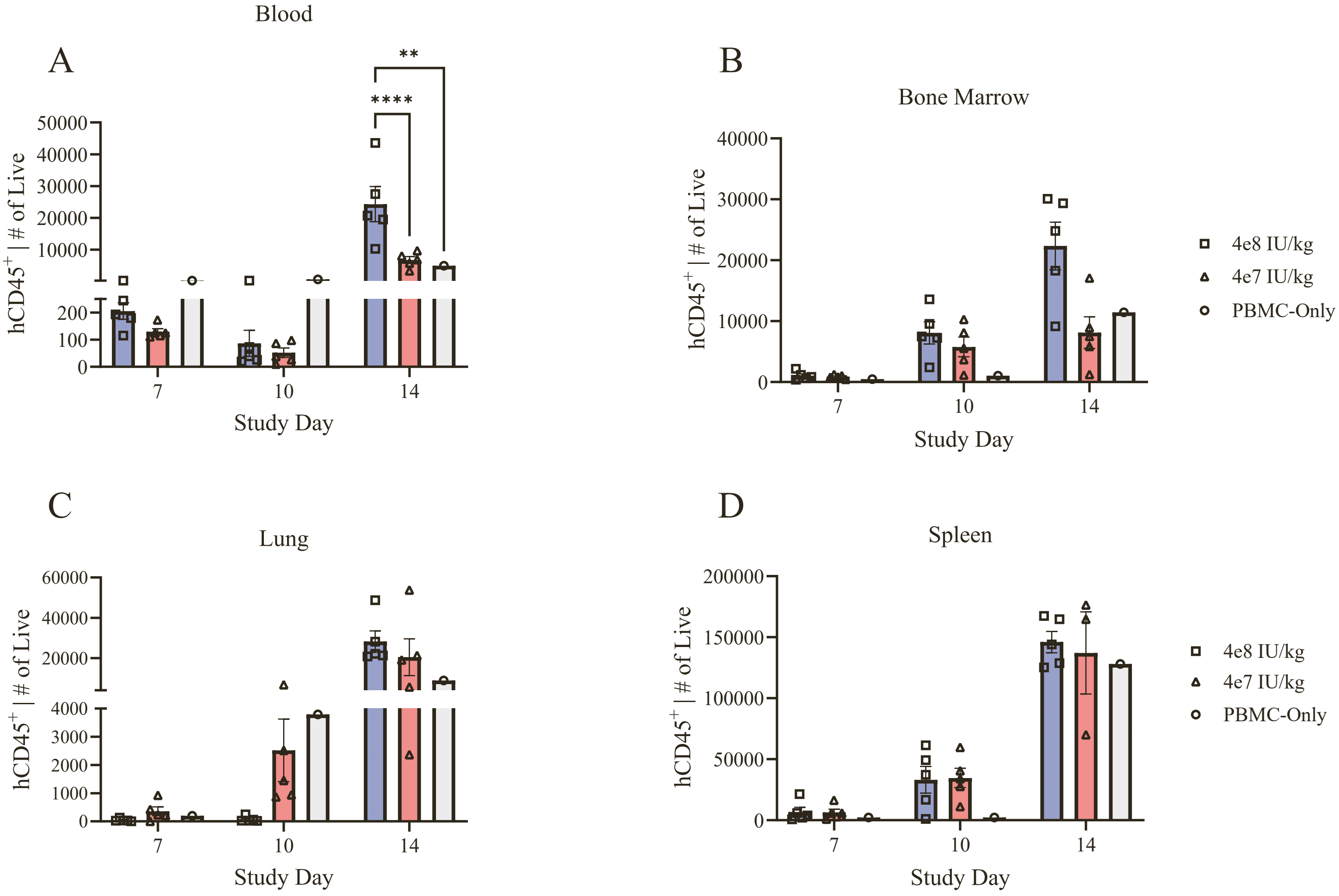

Blood
A
**
****
50000
40000
30000
20000
10000
200
100
0
hCD45+ | # of Live
7
10
14
Study Day
B
Bone Marrow
40000
30000
20000
10000
0
hCD45+ | # of Live
7
10
14
Study Day
4e8 IU/kg
4e7 IU/kg
PBMC-Only
C
Lung
60000
40000
20000
4000
3000
2000
1000
0
hCD45+ | # of Live
7
10
14
Study Day
D
Spleen
200000
150000
100000
50000
0
hCD45+ | # of Live
7
10
14
Study Day
4e8 IU/kg
4e7 IU/kg
PBMC-Only

Figure S1: Human CD45 Cell Detection in PBMC-engrafted Animals Over Time

A) Number of $CD45^+$ cells per 100 µl of blood over time in PBMC-alone (black), 4e8 IU/kg (blue), and 4e7 IU/kg (red) of LVV. **B)** Number of $CD45^+$ cells per 100 µl of bone marrow over time in PBMC-alone (black), 4e8 IU/kg (blue), and 4e7 IU/kg (red) of LVV. **C)** Number of $CD45^+$ cells per 100 µl of lung over time in PBMC-alone (black), 4e8 IU/kg (blue), and 4e7 IU/kg (red) of LVV. **D)** Number of $CD45^+$ cells per 100 µl of spleen over time in PBMC-alone (black), 4e8 IU/kg (blue), and 4e7 IU/kg (red) of LVV. Female NSG mice (n=5/sex/group) engrafted with human PBMCs on day -1, and LVV on day 0. The LOD of the assay is > 20 events. Statistical significance was measured by calculating a two-way ANOVA with a Bonferroni multiple comparison (*p<0.05, **p<0.01, ***p<0.005, ****p<0.001).

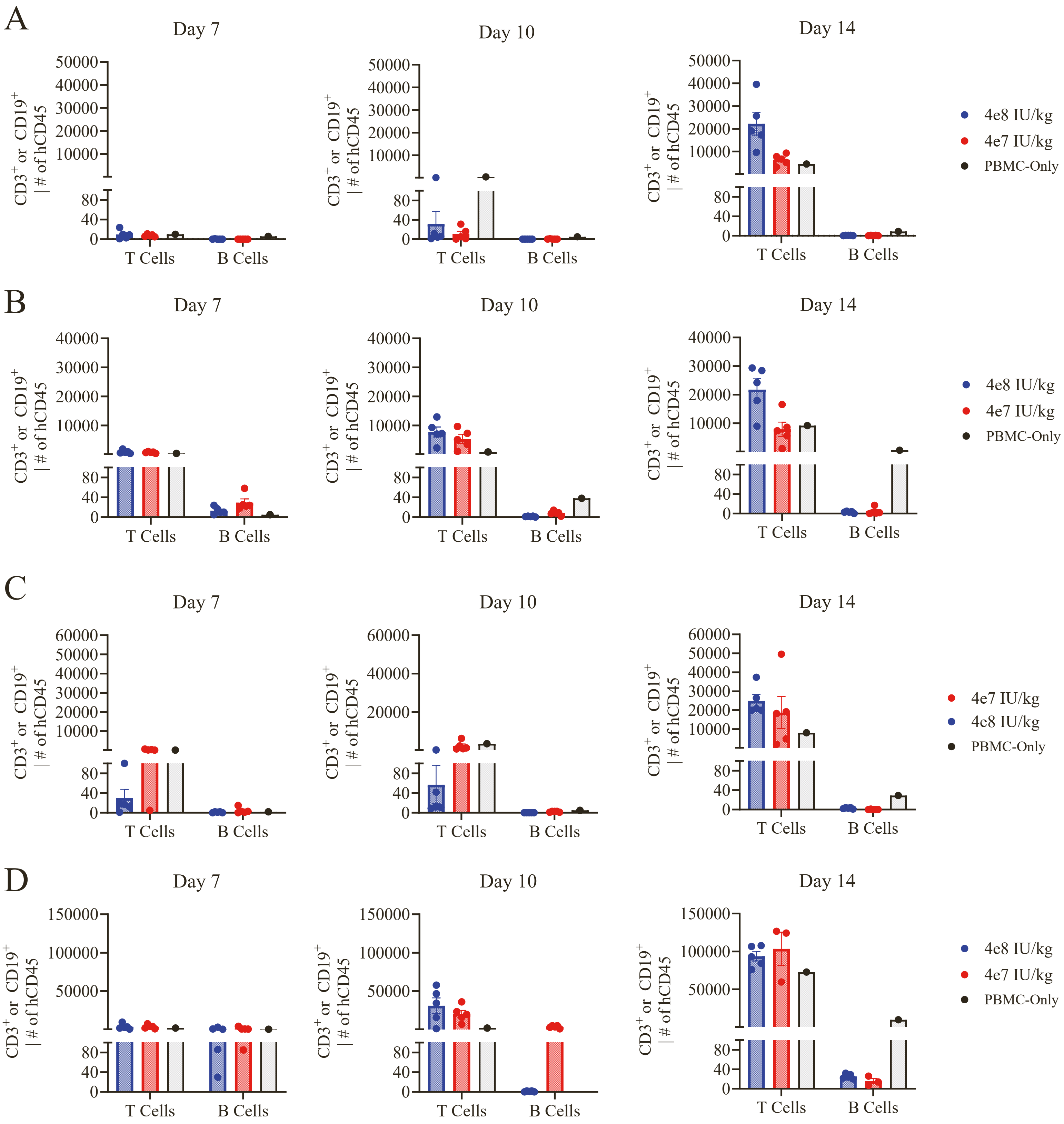

A
Day 7
Day 10
Day 14
$CD3^+$ or $CD19^+$ | # of hCD45
50000
40000
30000
20000
10000
80
40
0
T Cells
B Cells
4e8 IU/kg
4e7 IU/kg
PBMC-Only
B
40000
30000
20000
10000
C
60000
50000
D
150000
100000
50000

Figure S2: Human T Cell and B Cell Detection in PBMC-engrafted Animals Over Time

A) Number of $CD45^+$ $CD3^+$ and $CD19^+$ cells per 100 µl of blood on day 7, 10, and 14 in PBMC alone (black), 4e8 IU/kg (blue), and 4e7 IU/kg (red) of LVV. **B)** Number of $CD45^+$ $CD3^+$ and $CD19^+$ cells per 100 µl of bone marrow on day 7, 10, and 14 in PBMC-alone (black), 4e8 IU/kg (blue), and 4e7 IU/kg (red) of LVV. **C)** Number of $CD45^+$ $CD3^+$ and $CD19^+$ cells per 100 µl of lung on day 7, 10, and 14 in PBMC-alone (black), 4e8 IU/kg (blue), and 4e7 IU/kg (red) of LVV. **D)** Number of $CD45^+$ $CD3^+$ and $CD19^+$ cells per 100 µl of spleen on day 7, 10, and 14 in PBMC alone (black), 4e8 IU/kg (blue), and 4e7 IU/kg (red) of LVV. Female NSG mice (n=5/sex/group) engrafted with human PBMCs on day -1, and LVV on day 0. The LOD of the assay is > 20 events.

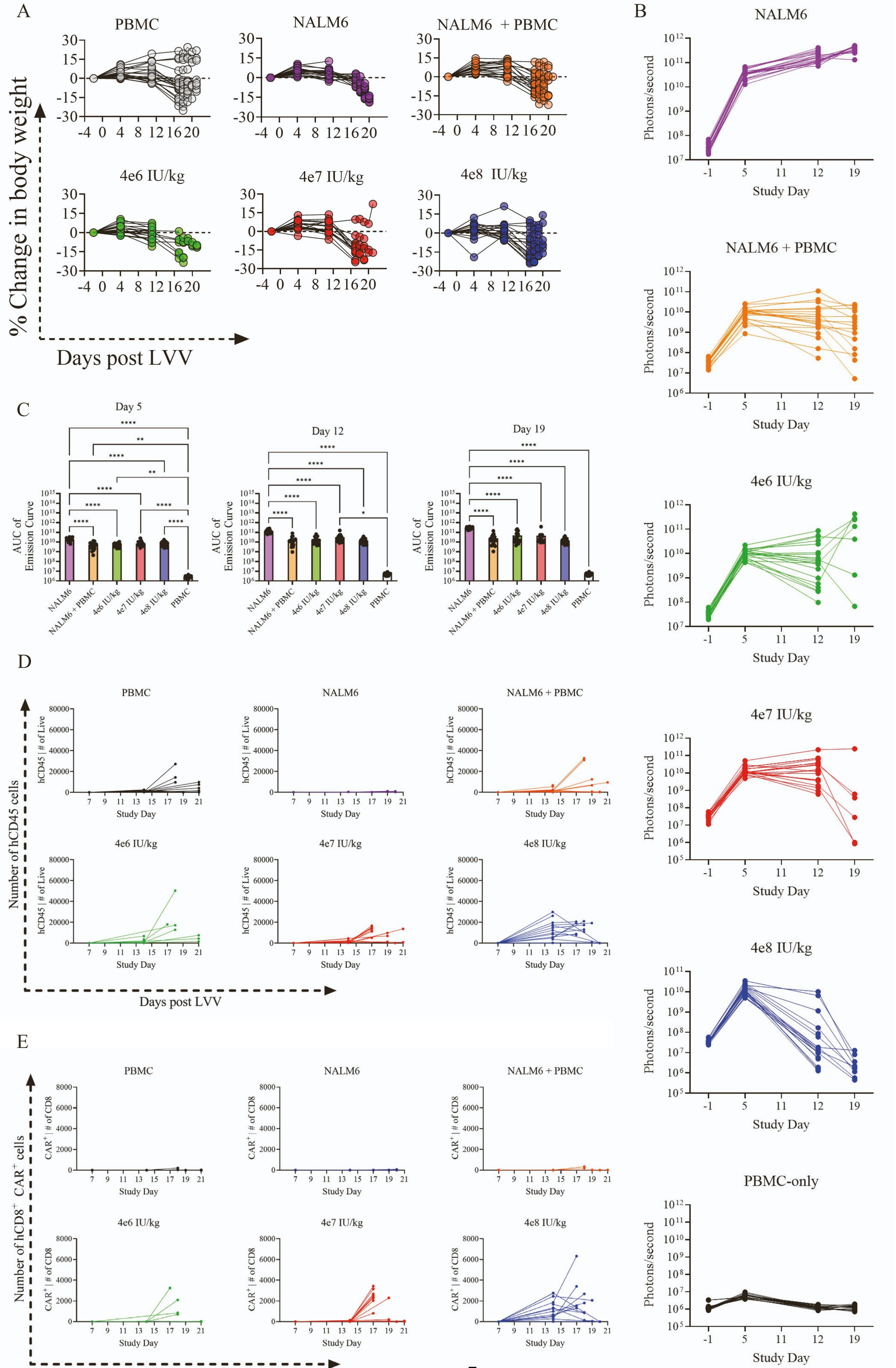

A
PBMC
NALM6
NALM6 + PBMC
4e6 IU/kg
4e7 IU/kg
4e8 IU/kg
% Change in body weight
Days post LVV
B
NALM6
NALM6 + PBMC
4e6 IU/kg
4e7 IU/kg
4e8 IU/kg
PBMC-only
Photons/second
Study Day
C
Day 5
Day 12
Day 19
AUC of Emission Curve
NALM6
NALM6 + PBMC
4e6 IU/kg
4e7 IU/kg
4e8 IU/kg
PBMC
D
hCD45 | # of Live
Study Day
Number of hCD45 cells
Days post LVV
E
CAR+ | # of CD8
Study Day
Number of hCD8+ CAR+ cells
Days post LVV

Figure S3: Pharmacodynamic Data from Donor 1

A) Percent change of body weight (from baseline measurement) over time in PBMC (black), NALM6 (purple), PBMC and NALM6 (orange), 4e6 IU/kg (green), 4e7 IU/kg (red), and 4e8 IU/kg (blue) of LVV. **B)** Photon per second values (tumor burden) graphed over time of individual animals that received either NALM6 (purple), PBMC and NALM6 (orange), 4e6 IU/kg (green), 4e7 IU/kg (red), 4e8 IU/kg (blue) of LVV or PBMC (black). **C)** Area under the curve of average photons per second (±SEM) values (tumor burden) graphed over time in PBMC (black), NALM6 (purple), PBMC and NALM6 (orange), 4e6 IU/kg (green), 4e7 IU/kg (red), and 4e8 IU/kg (blue) of LVV through day 5, 12, and 19. It is important to note that most animals were euthanized before the day 19 timepoint, so the data should be interpreted with caution. **D)** Number of $CD45^+$ cells per 100 µl of blood over time in PBMC (black), NALM6 (purple), PBMC and NALM6 (orange), 4e6 IU/kg (green), 4e7 IU/kg (red), and 4e8 IU/kg (blue) of LVV. The LOD of the assay is > 20 events. **E)** Number of $CD8^+CAR^+$ cells per 100 µl of blood over time in PBMC (black), NALM6 (purple), PBMC and NALM6 (orange), 4e6 IU/kg (green), 4e7 IU/kg (red), and 4e8 IU/kg (blue) of LVV. The LOD of the assay is > 20 events. Statistical significance was measured by calculating the area under the curve and measuring significance with a one-way ANOVA with a Bonferroni multiple comparison (*p<0.05, **p<0.01, ***p<0.005, ****p<0.001). Male and female NSG mice (n=5/sex/group) engrafted with $CD19^+$ NALM6 tumor cells on day -3, human PBMCs on day -1 from donor 1, and LVV on day 0.

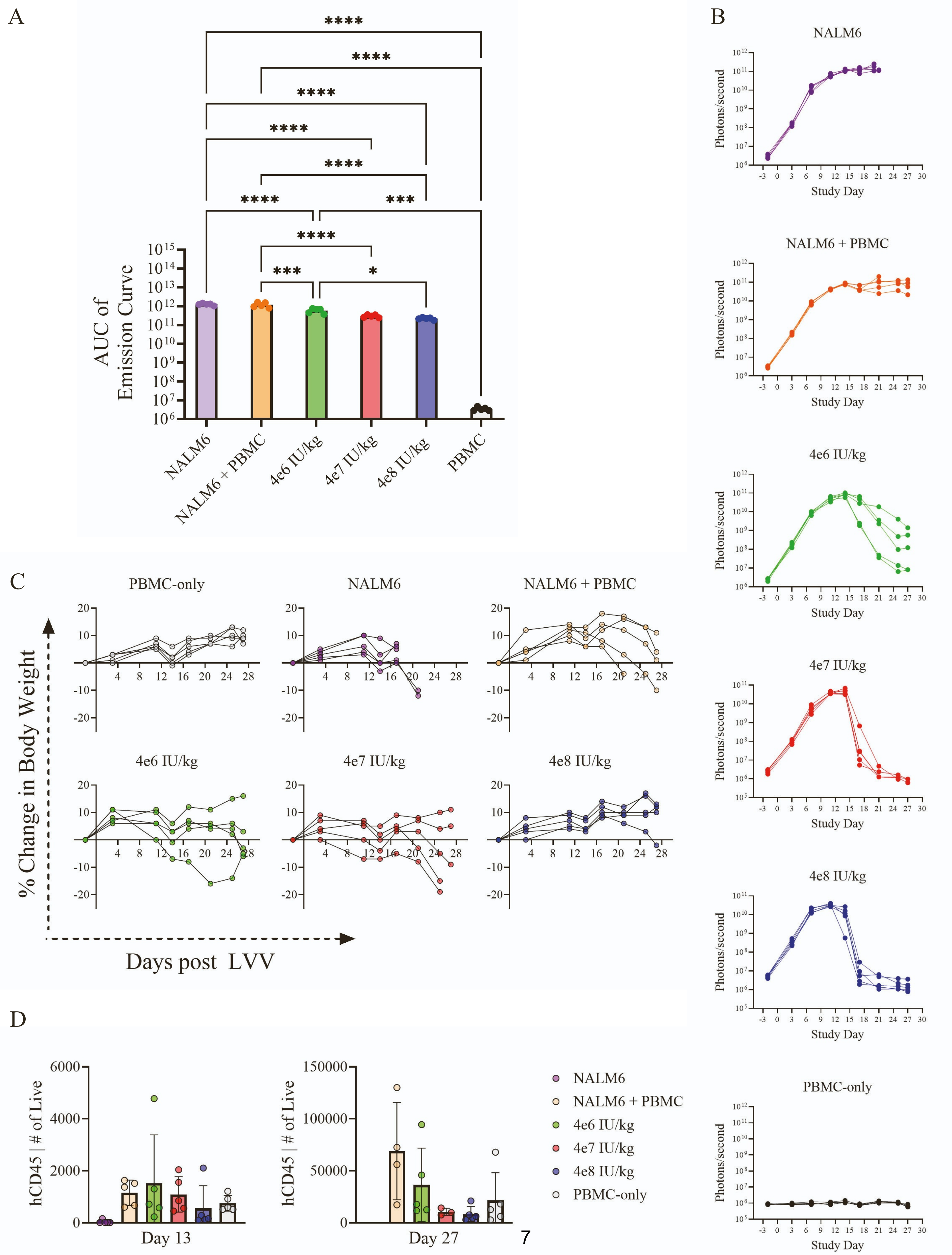

A
AUC of Emission Curve
NALM6
NALM6 + PBMC
4e6 IU/kg
4e7 IU/kg
4e8 IU/kg
PBMC
B
NALM6
NALM6 + PBMC
4e6 IU/kg
4e7 IU/kg
4e8 IU/kg
PBMC-only
Photons/second
Study Day
C
PBMC-only
NALM6
NALM6 + PBMC
4e6 IU/kg
4e7 IU/kg
4e8 IU/kg
% Change in Body Weight
Days post LVV
D
hCD45 | # of Live
Day 13
Day 27
NALM6
NALM6 + PBMC
4e6 IU/kg
4e7 IU/kg
4e8 IU/kg
PBMC-only

Figure S4: Pharmacodynamic Data from Donor 2

A) Area under the curve of average photons per second (±SEM) values (tumor burden) graphed over time in PBMC (black), NALM6 (purple), PBMC and NALM6 (orange), 4e6 IU/kg (green), 4e7 IU/kg (red), and 4e8 IU/kg (blue) of LVV over time. **B)** Photon per second values (tumor burden) graphed over time of individual animals that received either NALM6 (purple), PBMC and NALM6 (orange), 4e6 IU/kg (green), 4e7 IU/kg (red), 4e8 IU/kg (blue) of LVV or PBMC (black). **C)** Percent change of body weight (from baseline measurement) over time in PBMC (black), NALM6 (purple), PBMC and NALM6 (orange), 4e6 IU/kg (green), 4e7 IU/kg (red), and 4e8 IU/kg (blue) of LVV. **D)** Number of $CD45^+$ cells per 100 µl of blood over time in PBMC (black), NALM6 (purple), PBMC and NALM6 (orange), 4e6 IU/kg (green), 4e7 IU/kg (red), and 4e8 IU/kg (blue) of LVV. The LOD of the assay is > 20 events. Male and female NSG mice (n=5/sex/group) engrafted with $CD19^+$ NALM6 tumor cells on day -3, human PBMCs on day -1 from donor 2, and LVV on day 0. Statistical significance was measured by calculating the area under the curve and measuring significance with a one-way ANOVA with a Bonferroni multiple comparison (*p<0.05, **p<0.01, ***p<0.005, ****p<0.001).

Table S1. GQA Primer Sequences

Oligo Name	Primer and Probe Sequences
Viral Primer_Fwd	5'-GAGGAGGAGATATGAGGGACAA-3')
Viral Primer_Rev	5'-CACTCTTCTCTTTGCCTTGGT-3'
Viral Detection Probe	Fluorophore PrimeTime® 5`6-carboxyfluorescein (FAM™)<br>Non-fluorescent /ZENTM/and 3` Iowa Black® FQ(3`IABkFQ) double quencher:<br>5'- /5HEX/ATTGAACCA/ZEN/TTAGGAGTAGCACCC/3IABkFQ/-3'
mKate2_Fwd	5'-GTGGACAGAAGACTGGAAAGAA-3'
mKate2_Rev	5'-CTAGGGAGGTCGCAGTATCT-3'
mKate2 Detection Probe	Fluorophore PrimeTime ® 5`hexochlorofluorecein (HEX™)<br>Non-fluorescent /ZENTM/and 3` Iowa Black® FQ (3`IABkFQ) double quencher<br>5'-/5HEX/TCAAGGAGG/ZEN/CCGACAAAGAGACCTA/3IABkFQ/-3'

Table S2. VCN Primer Sequences

Oligo Name	Primer and Probe Sequence
CAR_Fwd	5'- CTCAAGAGGAAGATGGCTGTAG -3'
CAR_Rev	5'- CTGCTGAACTTCACTCTCAGT -3'
CAR_probe	5'- /56-FAM/CCTCCTTCT/ZEN/TCTTCTTCTGGAAATCGGC/3IABkFQ/- 3'
ARX_UCNE_Fwd	5'-TATGTTCAGATGCCCATTAGGG-3'
ARX_UCNE_Rev	5'-CTTGCTCAAAGGACTGTGATTTC-3'
ARX_UCNE_probe	5'-/5HEX/AGTGCCTTT/ZEN/CAGATGGAAACGGGT/3IABkFQ/-3'
